# Intraflagellar Transport Gene Expression Associated with Short Cilia in Smoking and COPD

**DOI:** 10.1371/journal.pone.0085453

**Published:** 2014-01-20

**Authors:** Justina Hessel, Jonna Heldrich, Jennifer Fuller, Michelle R. Staudt, Sharon Radisch, Charleen Hollmann, Ben-Gary Harvey, Robert J. Kaner, Jacqueline Salit, Jenny Yee-Levin, Sriram Sridhar, Sreekumar Pillai, Holly Hilton, Gerhard Wolff, Hans Bitter, Sudha Visvanathan, Jay Fine, Christopher S. Stevenson, Ronald G. Crystal, Ann E. Tilley

**Affiliations:** 1 Division of Pulmonary and Critical Care Medicine, Weill Cornell Medical College, New York, New York, United States of America; 2 Department of Genetic Medicine, Weill Cornell Medical College, New York, New York, United States of America; 3 Hoffmann-La Roche, Inc., Nutley, New Jersey, United States of America; VU University Medical Center, Netherlands

## Abstract

Smoking and COPD are associated with decreased mucociliary clearance, and healthy smokers have shorter cilia in the large airway than nonsmokers. We hypothesized that changes in cilia length are consistent throughout the airway, and we further hypothesized that smokers with COPD have shorter cilia than healthy smokers. Because intraflagellar transport (IFT) is the process by which cilia of normal length are produced and maintained, and alterations in IFT lead to short cilia in model organisms, we also hypothesized that smoking induces changes in the expression of IFT-related genes in the airway epithelium of smokers and smokers with COPD. To assess these hypotheses, airway epithelium was obtained via bronchoscopic brushing. Cilia length was assessed by measuring 100 cilia (10 cilia on each of 10 cells) per subject and Affymetrix microarrays were used to evaluate IFT gene expression in nonsmokers and healthy smokers in 2 independent data sets from large and small airway as well as in COPD smokers in a data set from the small airway. In the large and small airway epithelium, cilia were significantly shorter in healthy smokers than nonsmokers, and significantly shorter in COPD smokers than in both healthy smokers and nonsmokers. The gene expression data confirmed that a set of 8 IFT genes were down-regulated in smokers in both data sets; however, no differences were seen in COPD smokers compared to healthy smokers. These results support the concept that loss of cilia length contributes to defective mucociliary clearance in COPD, and that smoking-induced changes in expression of IFT genes may be one mechanism of abnormally short cilia in smokers. Strategies to normalize cilia length may be an important avenue for novel COPD therapies.

## Introduction

The lung is constantly exposed to inhaled particles, pathogens, irritants and toxins. In the normal lung, airway mucus provides a barrier to the airway epithelium and traps inhaled particles, which are removed from the airway via mucociliary clearance, the process by which cilia beat in a coordinated fashion to sweep mucus up out of the airways [1]–[3]. Impaired mucociliary clearance leads to a cycle of excessive airway mucus, recurrent pulmonary infection and worsening obstructive airway disease [4], [5]. In chronic obstructive pulmonary disease (COPD), mucociliary clearance is dysfunctional for a variety of reasons, which may include alterations in mucus composition and generation, and adverse effects of cigarette smoke on cilia structure and function [5], [6]. Prior work in our laboratory has shown that in the large airway epithelium (LAE, 2^nd^–5^th^ order airways), cigarette smoking in healthy individuals is associated with shorter cilia, with an observed difference in length that is likely to have an effect on mucociliary clearance [7].

How cilia length is controlled is not well understood. A number of theoretical mechanistic explanations have been proposed. These include the molecular ruler model, in which a protein with a physical length matching the cilia length controls cilia length [8], [9]; the limited precursor model, in which cilia length is limited by limited quantities of necessary precursor molecules [9], [10]; the cumulated strain model, in which cilia length is controlled by binding energy changes induced by changes in conformation with increasing length [9], [11]; a feedback control model, involving length sensing and signal transduction [9], [12]; and the balance point model, which is based on the knowledge that ciliary disassembly at the tip is ongoing at steady-state and appears to be length-independent, and which states that there is specific length at which this disassembly process is balanced with the rate of assembly, dependent on the rate of intraflagellar transport of components to the cilia tip [9], [13].

In addition to global mechanistic explanations, a number of specific genes have been implicated in length control. Some of these genes have been discovered through genetic screening of *Chlamydomonas* flagellar length mutants, including LF2, a cyclin-dependent kinase family member, and LF4, a mitogen-activated protein kinase family member [14]. In our previous report of shorter cilia in the large airway epithelium in healthy smokers, we found decreased expression of DNAH5, −10, and −11, as well as DYNC2H1, ezrin, and ODF2 in healthy smokers as compared to nonsmokers [7]. Consistent with the balance point model of length control, other investigators have specifically assayed proteins involved in intraflagellar transport, including those related to both the anterograde kinesin motor and the retrograde dynein 2 motor, as well as the intraflagellar transport genes IFT27, IFT46, IFT52, IFT70, IFT88 and IFT172 [13], [15]–[23], showing in model organisms that these genes are necessary for the production and/or maintenance of cilia of normal length.

We hypothesized that the changes in cilia length observed in the large airway of smokers are global throughout the airway, and that COPD smokers have shorter cilia than smokers without evidence of COPD. In exploring how smoking might impact cilia length, based on published observations of the central role of IFT in cilia length, we hypothesized that smoking causes decreased expression of intraflagellar transport genes in the human airway epithelium and that this reduced expression is associated with the shorter cilia observed in smokers. We addressed these hypotheses by assessing cilia length in the large and small airway epithelium of nonsmokers, healthy smokers, and smokers with COPD, and by quantifying expression of 40 IFT genes in the airway epithelium of these groups.

## Methods

### Ethics Statement

All individuals were evaluated and samples collected in the Weill Cornell NIH Clinical and Translational Science Center and Department of Genetic Medicine Clinical Research Facility under clinical protocols approved by the Weill Cornell Medical College and New York/Presbyterian Hospital Institutional Review Boards (IRB) according to local and national IRB guidelines. All subjects gave their informed written consent prior to any clinical evaluations or procedures.

### Study Population

Healthy nonsmokers, healthy smokers, and smokers with COPD were recruited from the general New York City population by placing advertisements in local newspapers and electronic bulletin boards. Individuals were determined to be phenotypically normal or to have COPD based on history, physical exam, complete blood count, coagulation studies, liver function tests, urine studies, chest X-ray, EKG, and pulmonary function tests. COPD was defined and staged according to the GOLD criteria [24]. Smoking status was confirmed with urine nicotine and cotinine levels. Full inclusion/exclusion criteria are detailed in Text S1. For COPD subjects, exacerbation frequency, Modified Medical Research Council dyspnea score [25], and St. George’s Respiratory Questionnaire scores [26] were assessed. High resolution chest CT was performed on a subset of subjects for quantitative analysis of emphysema (see Text S1). This study is registered under the ClinicalTrials.gov identifiers NCT00224185 and NCT00224198.

### Sample Collection and Preparation

All subjects underwent bronchoscopy for the acquisition of airway epithelial cells using standard methods [27]–[29]. Full details are available in Text S1.

### Cilia Length Analysis

Cilia length was assessed in the LAE and SAE of healthy nonsmokers, healthy smokers and COPD smokers in a randomized, blinded fashion. One slide was evaluated per subject. Each slide was visualized via bright field microscopy under 60x magnification using a Nikon Microphot-SE (Nikon, Melville, NY) and images of 10 ciliated cells per subject were taken using an Olympus DP-70 color CCD camera (Olympus, Center Valley, PA). Using Image J software (NIH, 2011), images were contrast enhanced and 10 cilia on each of 10 cells were measured for each subject. A mean cilia length was calculated for each individual. The average of all the means for each subject within a phenotype was taken for the group mean. Standard error of the mean and coefficient of variation were calculated for each individual and each phenotypic group. Further details regarding the method for determining mean cilia length and statistical analysis are provided in Text S1, Figure S1 and Table S1.

### Microarray Data Analysis

Microarray data were processed using the MAS5 algorithm (Affymetrix Microarray Suite Version 5 software) and GeneSpring software. Full details are available in Text S1. The raw microarray data are publically available at the Gene Expression Omnibus (GEO) site (http://www.ncbi.nlm.nih.gov/geo/) under the accession number GSE43939. Changes in the expression of significant genes were confirmed with TaqMan PCR; full details are available in Text S1.

## Results

### Study Population

Cilia length was assessed in 228 airway epithelium samples, including 120 LAE samples (n = 25 healthy nonsmokers, n = 25 healthy smokers, and n = 70 COPD smokers [n = 34 GOLD I, n = 29 GOLD II, n = 7 GOLD III]) and 108 SAE samples (n = 20 healthy nonsmokers, n = 32 healthy smokers, and n = 56 COPD smokers [n = 18 GOLD I, n = 29 GOLD II, n = 8 GOLD III, n = 1 GOLD IV]) (Table 1). Gene expression was assessed in the LAE of nonsmokers (n = 21) and healthy smokers (n = 31) and the SAE of an independent group of nonsmokers (n = 28) and healthy smokers (n = 69) as well as in the SAE of smokers with COPD (n = 59; n = 38 GOLD I, n = 20 GOLD II, n = 1 GOLD III) (Table 1). Both the healthy smokers and the smokers with COPD were generally older than the nonsmokers (gene expression set LAE p>0.1, SAE p<0.01; cilia length set LAE p<0.001, SAE p<0.003). Among subjects with LAE samples, healthy smokers and COPD smokers reported similar pack-yr of smoking (p>0.05); however, among subjects with SAE samples, smokers with COPD had higher reported pack-yr than healthy smokers (p<0.02 in the cilia length set and p<0.001 in the gene expression set). In the gene expression data set, among the LAE samples, there were no significant differences in the cell differentials between healthy smokers and nonsmokers (all p>0.1). Among the SAE samples, smokers had significantly fewer ciliated and basal cells and more undifferentiated cells compared to nonsmokers (all p<0.03); smokers with COPD had fewer ciliated cells than healthy smokers (p<0.02). In the cilia length data set, among LAE samples, COPD smokers had significantly more epithelial cells and fewer inflammatory cells than nonsmokers (both p<0.02), though all phenotypes had >98% epithelial cells. Healthy smokers and COPD smokers had fewer ciliated cells than nonsmokers, and COPD smokers had more secretory cells than nonsmokers and healthy smokers and more undifferentiated cells than nonsmokers (all p<0.05). Among the SAE samples, COPD smokers had fewer ciliated cells than nonsmokers and healthy smokers, more undifferentiated cells than nonsmokers, and more basal cells than healthy smokers (all p<0.04). In both the LAE and SAE groups smokers with COPD were slightly taller; this difference reached statistical significance (p<0.03) when compared to nonsmokers in the LAE group only. COPD smokers with GOLD II and III disease had significantly more emphysema on HRCT than those with GOLD I disease (5.1% *vs* 2.8%, p<0.02).

**Table 1:** Demographics^1^.

Parameter^1^	Gene expression^2^						Cilia length^3^					
	Large airway epithelium (n = 52)^4^		Small airway epithelium (n = 156)^5^				Large airway epithelium (n = 120)^4^			Small airway epithelium (n = 108)^5^		
	Healthynonsmokers	Healthysmokers	Healthynonsmokers	Healthysmokers		COPDsmokers	Healthynonsmokers	Healthysmokers	COPDsmokers	Healthynonsmokers	Healthysmokers	COPDsmokers
Number of subjects	21	31	28	69		59	25	25	70	20	32	56
Sex (male/female)	15/6	21/10	15/13	52/17		54/5	14/11	20/5	57/13	12/8	24/8	46/10
Age (yr)	41±8	44±6	39±12	46±9		54±8	38±11	49±5	53±8	40±11	48±5	54±9
Ethnicity (W/B/H/O)^6^	8/17/4/2	6/17/6/2	8/10/10/0	9/38/19/3		14/32/12/1	5/9/11/0	4/13/6/2	22/31/14/3	5/8/7/0	6/16/8/2	20/28/7/1
Height (inches)	69±4	67±3	67±3	68±4		70±3	67±3	68±4	69±4	67±3	68±4	69±4
Smoking history (pk-yr)	N/A	28±18	N/A	23±11		33±14	N/A	32.5±10	39±21	N/A	29±11	37±18
Pulmonary function^7^												
FEV_1_ (% predicted)	107±16	113±13	107±10	110±10		94±18	106±11	114±9	90±25	108±10	111±10	80±28
FVC (% predicted)	106±13	113±13	107±11	110±11		100±22	106±11	110±8	87±20	106±10	111±9	85±21
FEV1/FVC (% observed)	83±4	82±4	83±5	80±5		63±7	83±5	79±4	61±9	81±5	79±5	59±11
ΔFEV1 (L)^8^	–	–	–	–		0.17±0.15	–	–	0.16±0.17	–	–	0.20±0.16
ΔFEV1 (%)	–	–	–	–		6±6	–	–	6±7	–	–	6±5
ΔFVC (L)	–	–	–	–		0.17±0.67	–	–	0.12±0.21	–	–	0.13±0.24
ΔFVC (%)	–	–	–	–		2±5	–	–	4±8	–	–	3±6
TLC (% predicted)	100±13	103±12	99±15	94±15		101±13	100±16	100±11	99±13	100±14	100±13	95±12
RV/TLC (%)^9^	–	–	–	–		33±9	–	–	36±11	–	–	33±9
DLCO (% predicted)	94±12	95±11	91±12	88±8		71±15	89±10	90±8	69±17	89±10	90±8	68±18
DLCO/VA (% predicted)^10^	–	–	–	–		83±17	–	–	81±18	–	–	85±16
GOLD stage (I/II/III/IV)^11^	N/A	N/A	N/A	N/A		38/20/1/0	N/A	N/A	33/30/7/0	N/A	N/A	17/30/8/1
Exacerbations/yr	N/A	N/A	N/A	N/A		0.0±0.3	N/A	N/A	0.2±0.8	N/A	N/A	0.3±0.9
MMRC^12^	N/A	N/A	N/A	N/A		0.6±0.7	N/A	N/A	0.7±1.0	N/A	N/A	0.7±0.9
SGRQ^13^	N/A	N/A	N/A	N/A		21±16	N/A	N/A	23±17	N/A	N/A	22±17
HRCT %LAA^14^	*	*	1.8±1.8	1.3±1.0		3.6±3.4	2.2±1.9	1.3±0.9	3.3±2.8	2.2±1.9	1.5±1.0	3.7±3.6
Cell Differentials												
Epithelial (%)	99.8±0.5	99.8±0.4	99.0±0.9	99.2±0.8	97.5±7.8		98.6±1.1	99.1±0.9	99.2±0.9	98.8±0.8	98.6±1.2	96.1±9.3
Inflammatory (%)	0.2±0.5	0.2±0.4	0.9±0.8	0.7±0.8	2.5±7.8		1.4±1.1	0.9±0.9	1.8±0.9	1.2±0.7	1.4±1.2	3.9±9.3
Ciliated (%)	52.2±8.3	47.0±13.2	71.1±5.4	64.4±7.0	60.4±8.8		52.9±8.8	44.1±9.1	38.3±12.4	71.6±4.4	65.4±5.7	56.7±12.8
Secretory (%)	10.7±4.6	10.3±4.0	9.9±4.4	12.6±6.7	15.1±6.8		7.9±3.1	9.6±5.2	16.4±10.8	10.0±3.5	13.7±6.9	16.7±8.5
Undifferentiated (%)	16.0±8.8	18.0±10.0	7.5±3.6	14.3±8.2	13.6±7.2		12.4±6.7	19.7±11.3	22.4±13.1	7.2±2.7	11.8±5.5	12.4±6.5
Basal (%)	21.3±5.0	24.8±9.8	10.6±7.0	7.6±5.2	8.2±6.7		25.5±8.2	26.9±9.2	21.7±13.2	11.1±6.8	7.8±5.7	10.4±7.6

^1^ Values shown are mean ± standard deviation unless otherwise noted.

^2^ Data are shown for subjects who contributed samples for gene expression analysis.

^3^ Data are shown for subjects who contributed samples for cilia length assessment.

^4^ 3rd–4th order airway epithelium obtained by bronchoscopic brushing.

^5^ 10th–12th order airway epithelium obtained by bronchoscopic brushing.

^6^ Ethnicity is indicated as White (W), Black (B), Hispanic (H), Other (O).

^7^ Spirometry values shown are pre-bronchodilator. FEV1 =  forced expiratory volume in one second. FEV = forced vital capacity. TLC = total lung capacity. DLCO = diffusion capacity for carbon monoxide.

^8^ The response to bronchodilator is shown as change in volume and % change for FEV1 and FVC for COPD subjects.

^9^ The ratio of residual volume (RV) to total lung capacity (TLC) is shown as a % for COPD subjects.

^10^ The DLCO adjusted for alveolar volume (VA) is shown as % predicted for COPD subjects.

^11^ As defined by the Global Initiative for Obstructive Lung Disease criteria based on post-bronchodilator spirometry.

^12^ The Modified Medical Research Council dyspnea score ranges from 0–4 with higher scores reflecting a greater degree of dyspnea.

^13^ The St. George’s Respiratory Questionnaire measures the impact of COPD on overall health, daily life and well-being; scores range from 0–100 with higher scores = more limitations.

^14^% Low attenuation area is defined as the % of lung volume with attenuation values ≤ −950 Hounsfield units on quantitative analysis of HRCT.

* No CT data available for subjects in this group.

### Effect of Smoking and COPD on Cilia Length in the Large and Small Airway Epithelium

**Figure 1:**
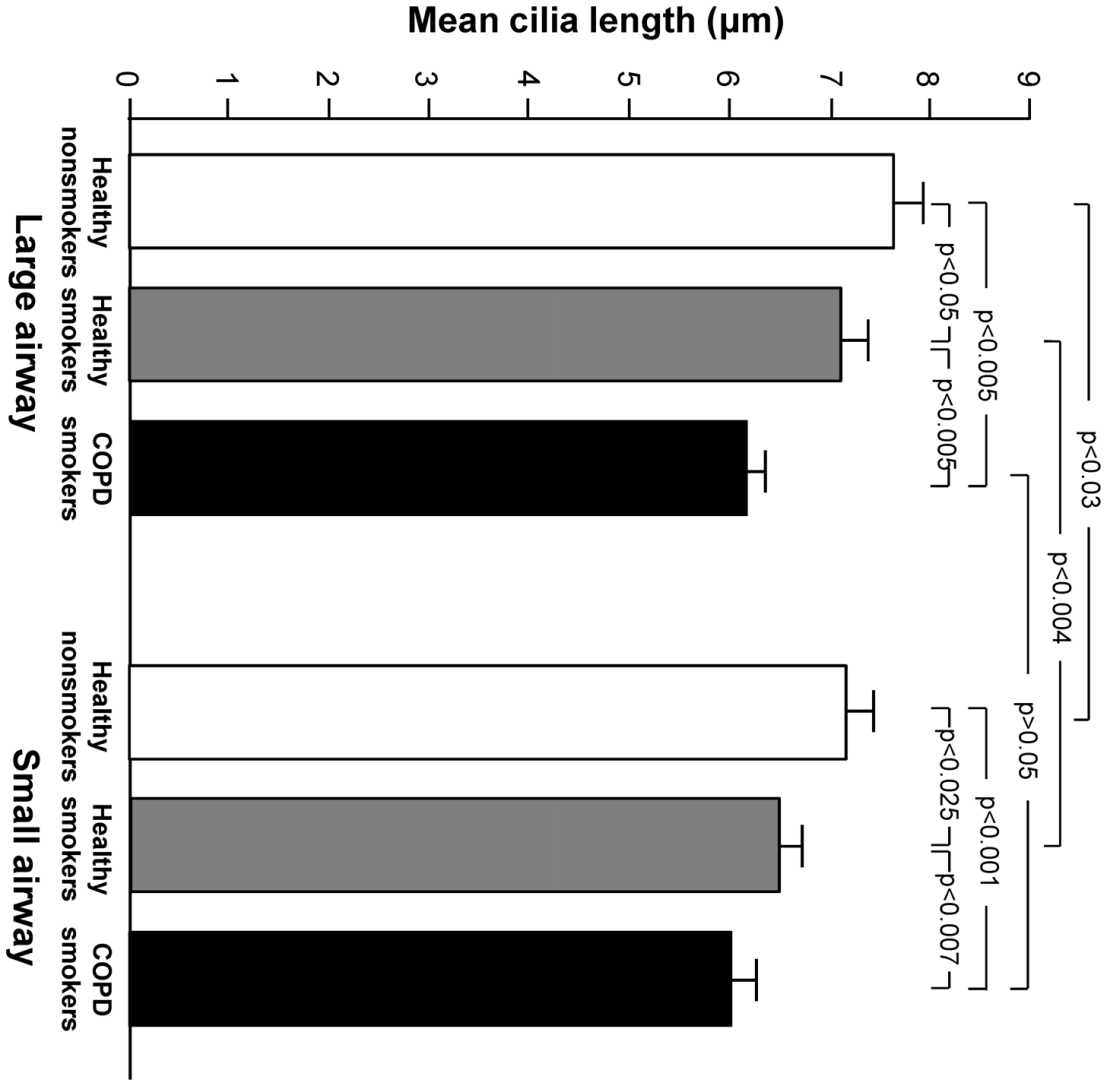
Cilia length in the large and small airway of nonsmokers, healthy smokers, and COPD smokers. Mean cilia length in micrometers is displayed on the ordinate. The abscissa displays large airway on the left and small airway on the right, with healthy nonsmokers represented in white, healthy smokers in dark gray, and smokers with COPD in black. Error bars show the standard error of the mean for each phenotype. Within the large airway, n = 120 (healthy nonsmokers n = 25, healthy smokers n = 25, smokers with COPD n = 70). Within the small airway, n = 108 (healthy nonsmokers n = 20, healthy smokers n = 32, smokers with COPD n = 56). There are significant differences in mean cilia length between all phenotypes in both the large and small airway (p<0.05). There are significant differences in mean cilia length between airway locations in both healthy nonsmokers and healthy smokers (p<0.05).

Cilia length was measured as described in Methods for nonsmokers, healthy smokers and COPD smokers (Figure S2). The method of measuring 100 cilia on one slide for each individual was used after determining that mean cilia length was not significantly different when 100 or 500 cilia were measured, and that intra-slide variability for one subject was less significant than intra-individual variability (see Text S1 and Table S1). Greater variability in cilia length for a given individual was observed among COPD smokers and healthy smokers than among nonsmokers (see Text S1 and Figure S3). The mean length for each phenotype was calculated as a mean of the individual means. In both the LAE and SAE, healthy smokers had shorter cilia than nonsmokers, and cilia were shorter in COPD smokers than in nonsmokers or healthy smokers (Figure 1). In the LAE, mean cilia length was 7% shorter in healthy smokers as compared to nonsmokers (7.09 *vs* 7.63 µm), 12% shorter in COPD smokers as compared to healthy smokers (6.16 *vs* 7.09 µm), and 19% shorter in COPD smokers as compared to nonsmokers. In the SAE, mean cilia length was 9% shorter in healthy smokers as compared to nonsmokers (6.49 *vs* 7.15 µm), 6% shorter in COPD smokers as compared to healthy smokers (6.05 *vs* 6.49 µm), and 15% shorter in COPD smokers as compared to nonsmokers. Mean cilia length was shorter in the SAE than in the LAE with significant differences observed in both nonsmokers (7.15 *vs* 7.63 µm) and healthy smokers (6.49 *vs* 7.09 µm), with a similar trend in COPD smokers (6.05 *vs* 6.16 µm).

To exclude an effect of age or sex on the findings, subsets of nonsmokers, healthy smokers and COPD smokers were chosen that were matched for age and sex. In the LAE, each group consisted of 4 women and 9 men and mean age was 47±7 for nonsmokers, 48±6 for healthy smokers, and 48±5 for COPD smokers (p>0.8). Mean cilia length was 7.8 µm for nonsmokers, 7.4 µm for healthy smokers, and 6.2 µm for COPD smokers (p<0.001 for COPD *vs* nonsmoker, p<0.002 for COPD *vs* smoker, but p>0.4 for smoker *vs* nonsmoker). In the SAE, each group consisted of 6 women and 9 men and mean age was 45±9 for nonsmokers, 47±6 for healthy smokers, and 47±6 for COPD smokers (p>0.5). Mean cilia length was 7.1 µm for nonsmokers, 6.6 µm for healthy smokers, and 5.9 µm for COPD smokers (p<0.001 for COPD *vs* nonsmoker, p<0.03 for COPD *vs* smoker, but p>0.1 for smoker *vs* nonsmoker).

Because the COPD smokers in the SAE group of the study reported significantly more pack-yr of smoking than did the healthy smokers, and the COPD smokers in the LAE group had a nonsignificant trend toward more pack-yr of smoking than healthy smokers, a subset of COPD smokers in each group was randomly selected that was matched for pack-yr with the healthy smokers. In the LAE, the matched subset of COPD smokers (n = 25) had a mean age of 49±5 compared to 49±5 for healthy smokers (n = 25, p>0.9) and mean pack-yr 33±11 *vs* 32±10 for healthy smokers (p>0.9). Mean cilia length for this subset of COPD smokers was 6.19 µm *vs* 7.09 µm for healthy smokers (p<0.001). Similarly, in the SAE, the matched subset of COPD smokers (n = 32) had a mean age of 50±7 yr, compared to 48±5 yr for the healthy smokers (n = 32, p>0.1) and a mean smoking history of 29±10 pack-yr, compared to a mean smoking history of 29±11 pack-yr for the healthy smokers (p>0.9). In this subset analysis, the mean cilia length remained significantly shorter in the COPD smokers: 6.01 µm *vs* 6.49 µm in healthy smokers (p<0.02).

### Clinical and Demographic Correlations with Cilia Length

**Figure 2:**
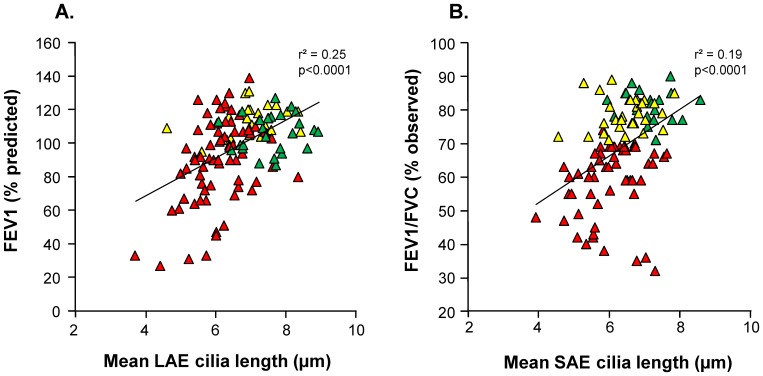
Correlation between mean cilia length and lung function. Each triangle represents one subject, with nonsmokers represented by green triangles, healthy smokers by yellow triangles, and COPD smokers by red triangles. **A.** Correlation of FEV1 with mean cilia length in large airway epithelium. The ordinate represents FEV1 (% predicted) and the abscissa represents mean cilia length in the LAE. **B.** Correlation of FEV1/FVC with mean cilia length in the small airway epithelium. The ordinate represents FEV1/FVC (% observed) and the abscissa represents mean cilia length in the SAE.

Correlations between mean cilia length and demographic and lung function parameters were assessed using univariate linear regression analysis (for continuous variables) and ANOVA (for categorical variables). In the LAE analysis, in the total population, there were significant correlations between cilia length and age, ethnicity, FEV1, FVC, FEV1/FVC, and DLCO (all p<0.01). When these factors were incorporated into a multivariate analysis, only FEV1, FVC, and FEV1/FVC remained as significant factors (all p<0.02). This analysis was repeated excluding nonsmokers and analyzing smokers (healthy and COPD) only. In the univariate analysis, significant factors were age, sex, ethnicity, FEV1, FEV1/FVC, and DLCO; in the multivariate analysis, only the FEV1 remained as a factor significantly correlated with mean cilia length (p<0.0001). In univariate analysis of FEV1 *vs* mean cilia length including all subjects, the r^2^ value was 0.25 (Figure 2A). COPD smokers with GOLD II-III disease had slightly shorter mean cilia length than those with GOLD I disease, but this difference was not significant (6.1 µm *vs* 6.2 µm, p>0.4).

Analysis was repeated in the same manner for the SAE. In the total population, significant correlations existed between cilia length and age, ethnicity, height, FEV1, FVC, FEV1/FVC, and DLCO (all p<0.03). In multivariate analysis, height and FEV1/FVC remained significant (both p<0.005). When only smokers were considered, significant correlations were found for age, sex, ethnicity, height, FEV1, FVC, and FEV1/FVC, but none of these correlations remained significant in multivariate analysis. In univariate analysis of FEV1/FVC in all subjects, the r^2^ value was 0.19 (Figure 2B). Consistent with the relationships observed between cilia length and spirometric variables, subjects with GOLD II-IV COPD had significantly shorter cilia than those with GOLD I disease (5.9 µm *vs* 6.4 µm, p<0.01).

### Effect of Smoking on Intraflagellar Transport Genes

**Figure 3:**
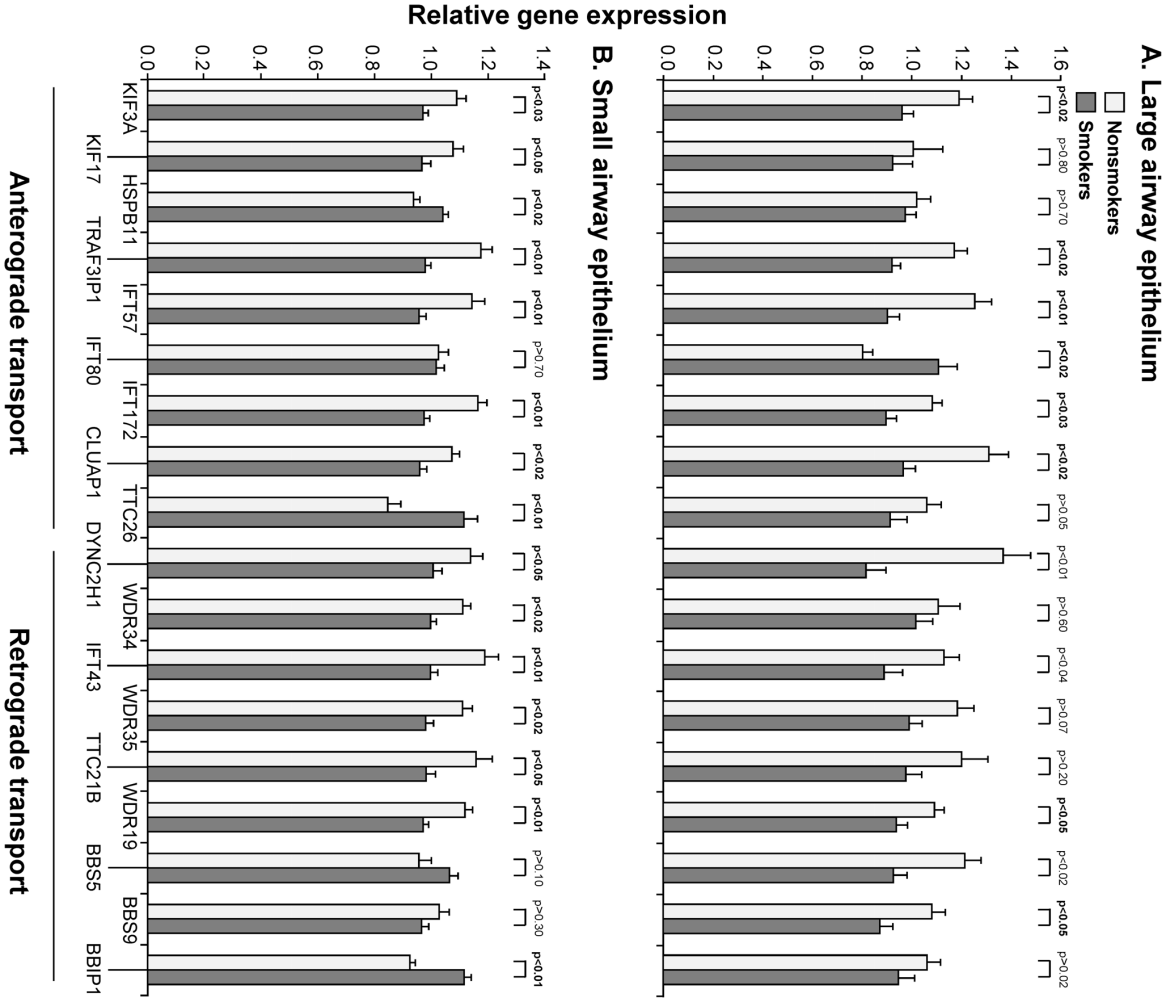
Expression of intraflagellar transport (IFT)-related genes significantly modified by smoking in the airway epithelium. Gene expression was assessed in nonsmokers and healthy smokers by microarray analysis in two independent data sets. The ordinate represents relative gene expression and specific genes are displayed on the abscissa. Nonsmokers are represented by light bars and healthy smokers by dark gray bars. Error bars represent standard error of the mean and p values are calculated using the Benjamini-Hochberg correction. Significant p values are displayed in bold font. Where multiple probe sets represent the same gene, the probe set with the lowest p value is displayed; where p values are identical, the probe set with highest fold-change is displayed. **A.** Expression in the large airway epithelium (nonsmokers n = 21 and healthy smokers n = 31). **B.** Expression in the small airway epithelium (nonsmokers n = 28 and healthy smokers n = 69).

To evaluate the hypothesis that smoking-induced changes in cilia length may be due, in part, to changes in the expression of genes encoding proteins relevant to intraflagellar transport, a list of 40 known, human IFT-related genes was compiled and expression of these genes evaluated in the LAE and SAE [30]. All 40 known IFT-related genes were expressed in both the LAE and SAE data sets (Table S2). Analysis of expression in smokers *vs* nonsmokers in the LAE revealed 13 probe sets representing 11 genes with expression significantly modified in smokers. Of these 11 genes, 10 were down-regulated and 1 (IFT80) was up-regulated in smokers. The down-regulated genes included a component of the anterograde IFT motor, and 4 genes involved in the IFT complex B (anterograde transport), including TRAF3IP1 (IFT54), IFT57, IFT172, and CLUAP1. DYNC2H1, a component of the retrograde transport motor, was down-regulated in smokers, as were 2 components of the IFT complex A (retrograde transport), IFT43 and WDR19 (IFT144). Two components of the BBSome, an IFT complex accessory, were also down-regulated, BBS5 and BBS9 (Figure 3A).

Expression analysis in the SAE revealed similar results. Sixteen probe sets representing 14 genes were significantly modified in smokers; of these, 3 were up-regulated (HSPB11/IFT25, TTC26, and BBIP1/BBIP10) and 11 down-regulated. Among the anterograde transport category, down-regulated genes were KIF3A and KIF17, as well as TRAF3IP1 (IFT54), IFT57, IFT172, and CLUAP1. Down-regulated genes also included the retrograde transport gene DYNC2H1, as well as another retrograde motor component, WDR34, and IFT43, WDR35 (IFT121), and WDR19 (IFT144; Figure 3B).

Combining these two analyses revealed a total of 8 IFT-related genes for which expression was similarly down-regulated by smoking in the independent data sets of LAE and SAE. These included 5 anterograde transport genes, KIF3A, TRAF3IP1/IFT54, IFT57, IFT172, and CLUAP1; and 3 retrograde transport genes, DYNC2H1, IFT43, and WDR19/IFT144. TaqMan PCR was carried out for significant genes on samples from the LAE and confirmed the changes observed by microarray (Table S3) with a significant p value for 5 of these genes.

### Effect of COPD Status on Intraflagellar Transport Genes

To assess whether the difference in mean cilia length in COPD smokers compared to healthy smokers could be due to further down-regulation of IFT genes in this group, we assessed IFT gene expression in COPD smokers compared to smokers without COPD. No significant differences in gene expression were found in COPD smokers *vs* healthy smokers for the 40 IFT genes assessed. We also assessed for differences in expression of these genes between GOLD I and GOLD II-III subjects and found no differences.

### Correlation of Gene Expression with Cilia Length

**Figure 4:**
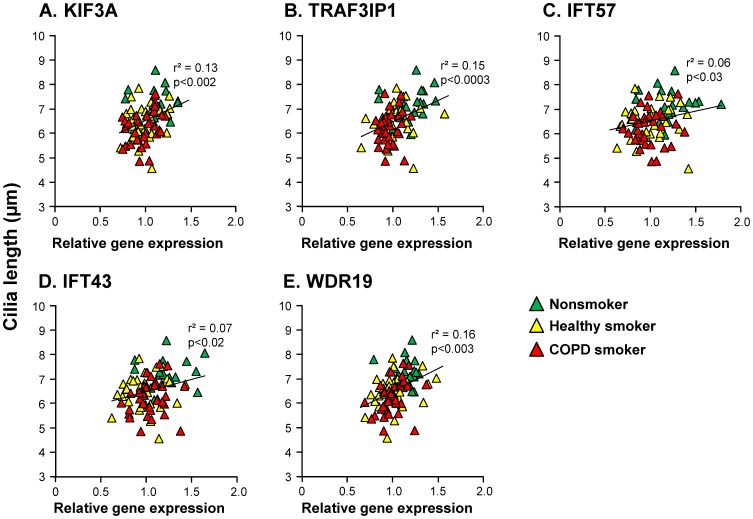
Correlation of cilia length with gene expression in the small airway epithelium. The ordinate represents mean cilia length for each individual and the abscissa represents relative gene expression. Each individual is represented by a triangle with nonsmokers represented by green triangles, healthy smokers by yellow triangles, and COPD smokers by red triangles. **A.** Correlation of cilia length with KIF3A expression. **B.** Correlation of cilia length with TRAF3IP1 expression. **C.** Correlation of cilia length with IFT57 expression. **D.** Correlation of cilia length with IFT43 expression. **E.** Correlation of cilia length with WDR19 expression.

Finally, we assessed whether the expression of the 8 IFT genes with smoking-induced down-regulation as confirmed in two independent data sets correlated with cilia length. This was done using the subset of subjects with SAE samples for whom both cilia length and gene expression were available (n = 83; there were no LAE subjects who had both gene expression and cilia length data). Genes were assessed first using univariate analysis. The results indicated that for 5 of the 8 genes, there was a modest but significant correlation between expression and cilia length: KIF3A, TRAF3IP1, IFT57, IFT43, and WDR19 (all p<0.05; Figure 4A–E). These 5 genes were then incorporated into a multivariate analysis and this model also significantly correlated with cilia length (p<0.005, r^2^ = 0.19).

## Discussion

Disorders of cilia are typically thought of as inherited disorders, the most prominent ciliopathy being primary ciliary dyskinesia, a clinical syndrome composed of pulmonary disease, male infertility, and, in 50% of patients, *situs inversus* [31], [32]. COPD can be thought of as an acquired, rather than inherited, ciliopathy in which cigarette smoke induces dysfunction in mucociliary clearance by a variety of mechanisms related both to abnormal mucus and mucin expression [5], [6], as well as to abnormal cilia structure and function [6], [33], [34]. This mucociliary dysfunction can be devastating, as mucus stasis leads to a vicious cycle of recurrent respiratory infections and worsening airflow obstruction [4], [5]. Proposed mechanisms of dysfunctional cilia in smokers include decreased numbers of ciliated cells, absent cilia structural components, changes in cilia beat frequency and coordination of ciliary beating, and altered mucus hydration status [34]–[37]. We have reported that healthy smokers have shorter cilia in the large airway than do nonsmokers [7]. In the current study we show that smokers with COPD have even shorter cilia than healthy smokers and that these changes are present in the distal as well as in the proximal airway, and we present data suggesting that smoking represses the expression of genes involved in intraflagellar transport, which may be at least partially causative of the short cilia, and that expression of several IFT-related genes correlates with cilia length.

Very few studies have addressed the effect of smoking on airway epithelial cilia length. In 1957, Chang [38] evaluated respiratory epithelium obtained at autopsy from the lungs of smokers with unknown lung function (n = 71) and nonsmokers (n = 34) and observed that smokers had shorter airway cilia than nonsmokers. Serafini and Michaelson [39] observed that cilia length decreases with increasing airway order in subjects without lung disease (but unknown smoking status) and described shorter cilia in a single subject with COPD. Tamashiro et al [40] found that exposure to cigarette smoke extract inhibited ciliogenesis and resulted in shorter cilia in cultured mouse nasal epithelium. Most recently, we reported that cilia in the large airway epithelium of clinically healthy smokers were significantly shorter than those of healthy nonsmokers [7].

To understand potential mechanisms for the shorter cilia observed in smokers and COPD smokers, we focused on intraflagellar transport (IFT), the process by which molecular motors transport and assemble tubulin at the distal ends of cilia [30]. IFT is required both for the growth and maintenance of normal cilia, as cilia maintenance is a dynamic process with ongoing turnover of tubulin at the distal tip necessitating transport of cilia proteins to the distal tip (anterograde IFT) and back to the cell body (retrograde IFT) [30]. Anterograde transport is driven by the kinesin-2 motor and utilizes proteins of IFT complex B. Retrograde transport is driven by the multiprotein cytoplasmic dynein 2 motor and utilizes proteins of IFT complex.

A number of proteins related to IFT have been shown in model systems to directly affect cilia length. Disruption of the anterograde kinesin motor results in short or absent cilia in a number of organisms [13], [30] as do alterations in the retrograde cytoplasmic dynein motor [15], [30]. Homologs of IFT27, IFT46, IFT52, IFT70, IFT88 and IFT172, all relevant to anterograde transport, as well as the BBSome component BBS9, have been demonstrated in model organisms to be necessary for generating and/or maintaining cilia of normal length [16]–[23], [41]. Less is known about genetic control of cilia length in humans. Interestingly, however, members of a consanguineous family with short-rib polydactyly syndrome were found to have mutations in the dynein heavy chain DYNC2H1 resulting in decreased mRNA expression, and cultured chondrocytes from these individuals demonstrated short cilia and cells with absent cilia [42]. In our previous study of cilia length in the large airway of smokers, DYNC2H1 was down-regulated in the healthy smokers [7]. Further, members of a family with Sensenbrenner syndrome, a ciliopathy characterized by abnormal bony development as well as frequent extraskeletal abnormalities, were found to have a mutation in WDR19 (encoding IFT144); cultured fibroblasts from a Sensenbrenner patient also displayed reduced cilia length compared to cilia from fibroblasts from control cell lines [43].

To expand on our observation of shorter cilia in the LAE of healthy smokers, we sought to address two questions: (1) how does the cilia length of smokers with COPD compare to that of nonsmokers and healthy smokers; and (2) are these changes also seen in the small airway, the earliest site of abnormality in the development of COPD? The data indicate that the cilia of COPD smokers are 6 to 12% shorter than the cilia of smokers without COPD and 15 to 19% shorter than the cilia of healthy nonsmokers. This reduction in length would be expected to significantly adversely affect mucociliary clearance, both by reducing the ability of the cilia tips to make contact with the mucus layer as well as by reducing the force generated on the forward stroke of the cilia, and provides an additional mechanism by which mucociliary clearance is impaired in smokers with COPD. Our results also show that smokers and COPD smokers have shorter cilia in both the large (3^rd^–4^th^ order) and small (10^th^–12^th^) order airways. Statistically significant correlations were observed between mean cilia length and physiologic markers of airflow obstruction, FEV1 in the LAE analysis and FEV1/FVC in the SAE analysis, suggesting that cilia length may play a role in the pathogenesis of disease in smokers. In the SAE analysis, a significant correlation was also found for height. The significance of this is uncertain, and the degree of correlation was small (r^2^ = 0.05); this is unlikely to have influenced our results, as COPD subjects in our study were on average slightly taller than nonsmokers.

To assess potential mechanisms for the observed differences in cilia length, we hypothesized that smoking down-regulates expression of IFT genes in the airway epithelium and used microarray technology to assay the expression of 40 human IFT genes representing both anterograde IFT (the kinesin-2 motor and IFT complex B) and retrograde IFT (the cytoplasmic dynein 2 motor, IFT complex A) as well as the BBSome in the human airway epithelium in smokers and nonsmokers in two independent data sets representing large and small airway epithelium. The results indicated 8 IFT genes with expression modulated by smoking as confirmed in the two independent data sets. Among genes involved in anterograde transport, 5 were significantly down-regulated in association with smoking in both data sets. These included both genes for which the literature supports a role in maintenance of cilia length (KIF3A [13] and IFT172 [23]), and genes for which no specific role in cilia length maintenance has been elucidated (IFT54, IFT57, and CLUAP1). Similarly, among the 3 down-regulated retrograde transport genes, there is data supporting the role of DYNC2H1 and IFT144 in cilia length [15], [30], [42], [43], and we have previously reported on decreased DYNC2H1 expression in healthy smokers with short cilia [7], but no specific role in cilia length maintenance has been reported for IFT43. In the context that smoking, in general, both up- and down-regulates large numbers of genes in the airway epithelium [27]–[29], [44]–[46], with some categories of genes, such as oxidant-related genes, being strongly up-regulated by smoking, the finding of down-regulation of IFT genes supports the concept that there is a specific effect of smoking on IFT. The mechanism by which smoking affects both anterograde and retrograde IFT genes is an important area for future study. One potential mechanism is smoking-induced suppression of a common cilia-related transcription factor, such as FOXJ1. However, other mechanisms, such as epigenetic modification, may be involved, and the present study does not answer this question.

Interestingly, despite the finding of shorter cilia in COPD smokers as compared to healthy smokers, even when matched for pack-yr, no differences in IFT expression were observed between these groups. The present study does not explain why this is the case. Given the complexity of cilia assembly and maintenance, and the many effects of cigarette smoke on the airway epithelium, this finding may suggest that there are additional mechanisms aside from alterations in IFT contributing to cilia length control in smokers. Alternatively, given the variability in cilia length observed among individuals, it may suggest that those smokers with the shortest cilia are at increased risk for the development of COPD.

The role of cilia in cells is diverse and is not limited to a clearance function. Emerging data suggests a role for motile cilia in cell signaling and IFT proteins may play a direct role in several signaling pathways [47]. An intriguing area for future study is the effect of smoking on the signaling function of airway cilia.

These results support the concept that loss of normal cilia length may contribute importantly to the defective mucociliary clearance seen in COPD. The fact that abnormally short cilia are also found in the small airways of healthy smokers and COPD smokers is consistent with the obstruction and dysfunctional mucociliary clearance seen in the small airways in COPD [4], [5]. In conjunction with the findings of short cilia in large and small airways of healthy and COPD smokers, we have found that 8 genes involved in IFT are down-regulated in the airway epithelium in association with smoking, and the expression of 5 of these genes correlates with cilia length. These results do not prove causation; such studies, for instance evaluating the effect of knocking down expression of IFT genes in human cells, would be very difficult given the multiple genes involved in IFT with probable overlapping function. However, while these results must be interpreted with caution, the inclusion in this list of 4 genes with known or suggested roles in maintenance of normal cilia length adds strength to the findings. It is likely that the effect of smoking on IFT is complex and involves the suppression of a number of key IFT genes, which may be one mechanism of abnormally short cilia in healthy and diseased smokers. Further study of the process by which smoking results in shorter cilia in smokers and of how these shorter cilia impact mucociliary clearance should lead to novel therapeutic avenues targeted at the dysfunctional mucociliary clearance that is a key feature of COPD.

## Supporting Information

Figure S1 Cilia length distributions obtained by measuring 100 cilia on 10 cells *vs* 500 cilia on 50 cells for each individual.
(PDF)

Figure S2 Example of determination of mean cilia length in one individual.
(PDF)

Figure S3 Variability of cilia length in the large (LAE) and small airway epithelium (SAE) of nonsmokers, healthy smokers and COPD smokers.
(PDF)

Table S1 Inter-slide *vs* Inter-individual Variability in Cilia Length.
(PDF)

Table S2 Expression of Intraflagellar Transport Genes in the Large and Small Airway Epithelium.
(PDF)

Table S3 TaqMan Confirmation of Significantly Modified Intraflagellar Transport Genes.
(PDF)

Text S1 Supplemental Methods.
(PDF)

## References

[1] Wanner A, Salathe M, O’Riordan TG (1996) Mucociliary clearance in the airways. Am J Respir Crit Care Med 154: 1868–1902. 8970383 10.1164/ajrccm.154.6.8970383

[2] Houtmeyers E, Gosselink R, Gayan-Ramirez G, Decramer M (1999) Regulation of mucociliary clearance in health and disease. Eur Respir J 13: 1177–1188. 10414423 10.1034/j.1399-3003.1999.13e39.x

[3] Button B, Cai LH, Ehre C, Kesimer M, Hill DB, et al (2012) A periciliary brush promotes the lung health by separating the mucus layer from airway epithelia. Science 337: 937–941. 22923574 10.1126/science.1223012 PMC3633213

[4] Hogg JC (2004) Pathophysiology of airflow limitation in chronic obstructive pulmonary disease. Lancet 364: 709–721. 15325838 10.1016/S0140-6736(04)16900-6

[5] Fahy JV, Dickey BF (2010) Airway mucus function and dysfunction. N Engl J Med 363: 2233–2247. 21121836 10.1056/NEJMra0910061 PMC4048736

[6] Voynow JA, Rubin BK (2009) Mucins, mucus, and sputum. Chest 135: 505–512. 19201713 10.1378/chest.08-0412

[7] Leopold PL, O’Mahony MJ, Lian XJ, Tilley AE, Harvey BG, et al (2009) Smoking is associated with shortened airway cilia. PLoS One 4: e8157 10.1371/journal.pone.0008157 20016779 PMC2790614

[8] Fowler VM, McKeown CR, Fischer RS (2006) Nebulin: does it measure up as a ruler? Curr Biol 16: R18–R20. 16401411 10.1016/j.cub.2005.12.003

[9] Wemmer KA, Marshall WF (2007) Flagellar length control in chlamydomonas–paradigm for organelle size regulation. Int Rev Cytol 260: 175–212. 17482906 10.1016/S0074-7696(06)60004-1

[10] Norrander JM, Linck RW, Stephens RE (1995) Transcriptional control of tektin A mRNA correlates with cilia development and length determination during sea urchin embryogenesis. Development 121: 1615–1623. 7600979 10.1242/dev.121.6.1615

[11] Wagenknecht T, Bloomfield VA (1975) Equilibrium mechanisms of length regulation in linear protein aggregates. Biopolymers 14: 2297–2309.

[12] Rosenbaum J (2003) Organelle size regulation: length matters. Curr Biol 13: R506–R507. 12842024 10.1016/s0960-9822(03)00440-8

[13] Marshall WF, Qin H, Rodrigo BM, Rosenbaum JL (2005) Flagellar length control system: testing a simple model based on intraflagellar transport and turnover. Mol Biol Cell 16: 270–278. 15496456 10.1091/mbc.E04-07-0586 PMC539171

[14] Avasthi P, Marshall WF (2012) Stages of ciliogenesis and regulation of ciliary length. Differentiation 83: S30–S42. 22178116 10.1016/j.diff.2011.11.015 PMC3269565

[15] Rajagopalan V, Subramanian A, Wilkes DE, Pennock DG, Asai DJ (2009) Dynein-2 affects the regulation of ciliary length but is not required for ciliogenesis in Tetrahymena thermophila. Mol Biol Cell 20: 708–720. 19019986 10.1091/mbc.E08-07-0746 PMC2626569

[16] Qin H, Wang Z, Diener D, Rosenbaum J (2007) Intraflagellar transport protein 27 (IFT27) is a small G protein involved in the control of cell division. Curr Biol 17: 193–202. 17276912 10.1016/j.cub.2006.12.040 PMC1905864

[17] Hou Y, Qin H, Follit JA, Pazour GJ, Rosenbaum JL, et al (2007) Functional analysis of an individual IFT protein: IFT46 is required for transport of outer dynein arms into flagella. J Cell Biol 176: 653–665. 17312020 10.1083/jcb.200608041 PMC2064023

[18] Brazelton WJ, Amundsen CD, Silflow CD, Lefebvre PA (2001) The bld1 mutation identifies the Chlamydomonas osm-6 homolog as a gene required for flagellar assembly. Curr Biol 11: 1591–1594. 11676919 10.1016/s0960-9822(01)00485-7

[19] Brown JM, Fine NA, Pandiyan G, Thazhath R, Gaertig J (2003) Hypoxia regulates assembly of cilia in suppressors of Tetrahymena lacking an intraflagellar transport subunit gene. Mol Biol Cell 14: 3192–3207. 12925756 10.1091/mbc.E03-03-0166 PMC181560

[20] Deane JA, Cole DG, Seeley ES, Diener DR, Rosenbaum JL (2001) Localization of intraflagellar transport protein IFT52 identifies basal body transitional fibers as the docking site for IFT particles. Curr Biol 11: 1586–1590. 11676918 10.1016/s0960-9822(01)00484-5

[21] Fan ZC, Behal RH, Geimer S, Wang Z, Williamson SM, et al (2010) Chlamydomonas IFT70/CrDYF-1 is a core component of IFT particle complex B and is required for flagellar assembly. Mol Biol Cell 21: 2696–2706. 20534810 10.1091/mbc.E10-03-0191 PMC2912355

[22] Pazour GJ, Dickert BL, Vucica Y, Seeley ES, Rosenbaum JL, et al (2000) Chlamydomonas IFT88 and its mouse homologue, polycystic kidney disease gene tg737, are required for assembly of cilia and flagella. J Cell Biol 151: 709–718. 11062270 10.1083/jcb.151.3.709 PMC2185580

[23] Tsao CC, Gorovsky MA (2008) Different effects of Tetrahymena IFT172 domains on anterograde and retrograde intraflagellar transport. Mol Biol Cell 19: 1450–1461. 18199688 10.1091/mbc.E07-05-0403 PMC2291410

[24] Global strategy for the diagnosis, management, and prevention of COPD, global initiative for chronic obstructive pulmonary disease (GOLD) 2011. Available: http://www.goldcopd.org/.

[25] Bestall JC, Paul EA, Garrod R, Garnham R, Jones PW, et al (1999) Usefulness of the Medical Research Council (MRC) dyspnoea scale as a measure of disability in patients with chronic obstructive pulmonary disease. Thorax 54: 581–586. 10377201 10.1136/thx.54.7.581 PMC1745516

[26] Barr JT, Schumacher GE, Freeman S, LeMoine M, Bakst AW, et al (2000) American translation, modification, and validation of the St. George’s Respiratory Questionnaire. Clin Ther 22: 1121–1145. 11048909 10.1016/S0149-2918(00)80089-2

[27] Hackett NR, Heguy A, Harvey BG, O’Connor TP, Luettich K, et al (2003) Variability of antioxidant-related gene expression in the airway epithelium of cigarette smokers. Am J Respir Cell Mol Biol 29: 331–343. 12702543 10.1165/rcmb.2002-0321OC

[28] Harvey BG, Heguy A, Leopold PL, Carolan BJ, Ferris B, et al (2007) Modification of gene expression of the small airway epithelium in response to cigarette smoking. J Mol Med (Berl) 85: 39–53. 17115125 10.1007/s00109-006-0103-z

[29] Tilley AE, O’Connor TP, Hackett NR, Strulovici-Barel Y, Salit J, et al (2011) Biologic phenotyping of the human small airway epithelial response to cigarette smoking. PLoS One 6: e22798 10.1371/journal.pone.0022798 21829517 PMC3145669

[30] Ishikawa H, Marshall WF (2011) Ciliogenesis: building the cell’s antenna. Nat Rev Mol Cell Biol 12: 222–234. 21427764 10.1038/nrm3085

[31] Hildebrandt F, Benzing T, Katsanis N (2011) Ciliopathies. N Engl J Med 364: 1533–1543. 21506742 10.1056/NEJMra1010172 PMC3640822

[32] Leigh MW, Pittman JE, Carson JL, Ferkol TW, Dell SD, et al (2009) Clinical and genetic aspects of primary ciliary dyskinesia/Kartagener syndrome. Genet Med 11: 473–487. 19606528 10.1097/GIM.0b013e3181a53562 PMC3739704

[33] Auerbach O, Hammond EC, Garfinkel L (1979) Changes in bronchial epithelium in relation to cigarette smoking, 1955–1960 vs. 1970–1977. N Engl J Med 300: 381–385. 759914 10.1056/NEJM197902223000801

[34] Verra F, Escudier E, Lebargy F, Bernaudin JF, De CH, et al (1995) Ciliary abnormalities in bronchial epithelium of smokers, ex-smokers, and nonsmokers. Am J Respir Crit Care Med 151: 630–634. 7881648 10.1164/ajrccm/151.3_Pt_1.630

[35] Fox B, Bull TB, Oliver TN (1983) The distribution and assessment of electron-microscopic abnormalities of human cilia. Eur J Respir Dis Suppl 127: 11–18. 6578054

[36] Ballenger JJ (1960) Experimental effect of cigarette smoke on human respiratory cilia. N Engl J Med 263: 832–835. 13686385 10.1056/NEJM196010272631704

[37] Fox B, Bull TB, Makey AR, Rawbone R (1981) The significance of ultrastructural abnormalities of human cilia. Chest 80: 796–799. 7307613 10.1378/chest.80.6.796

[38] Chang SC (1957) Microscopic properties of whole mounts and sections of human bronchial epithelium of smokers and nonsmokers. Cancer 10: 1246–1262. 13489677 10.1002/1097-0142(195711/12)10:6<1246::aid-cncr2820100623>3.0.co;2-z

[39] Serafini SM, Michaelson ED (1977) Length and distribution of cilia in human and canine airways. Bull Eur Physiopathol Respir 13: 551–559. 912142

[40] Tamashiro E, Xiong G, Anselmo-Lima WT, Kreindler JL, Palmer JN, et al (2009) Cigarette smoke exposure impairs respiratory epithelial ciliogenesis. Am J Rhinol Allergy 23: 117–122. 19401033 10.2500/ajra.2009.23.3280

[41] Veleri S, Bishop K, le Nogare DE, English MA, Foskett TJ, et al (2012) Knockdown of Bardet-Biedl syndrome gene BBS9/PTHB1 leads to cilia defects. PLoS One 7: e34389 10.1371/journal.pone.0034389 22479622 PMC3315532

[42] Merrill AE, Merriman B, Farrington-Rock C, Camacho N, Sebald ET, et al (2009) Ciliary abnormalities due to defects in the retrograde transport protein DYNC2H1 in short-rib polydactyly syndrome. Am J Hum Genet 84: 542–549. 19361615 10.1016/j.ajhg.2009.03.015 PMC2667993

[43] Bredrup C, Saunier S, Oud MM, Fiskerstrand T, Hoischen A, et al (2011) Ciliopathies with skeletal anomalies and renal insufficiency due to mutations in the IFT-A gene WDR19. Am J Hum Genet 89: 634–643. 22019273 10.1016/j.ajhg.2011.10.001 PMC3213394

[44] Gower AC, Steiling K, Brothers JF, Lenburg ME, Spira A (2011) Transcriptomic studies of the airway field of injury associated with smoking-related lung disease. Proc Am Thorac Soc 8: 173–179. 21543797 10.1513/pats.201011-066MS PMC3159071

[45] Pierrou S, Broberg P, O’Donnell RA, Pawlowski K, Virtala R, et al (2007) Expression of genes involved in oxidative stress responses in airway epithelial cells of smokers with chronic obstructive pulmonary disease. Am J Respir Crit Care Med 175: 577–586. 17158281 10.1164/rccm.200607-931OC

[46] Hubner RH, Schwartz JD, De BP, Ferris B, Omberg L, et al (2009) Coordinate control of expression of Nrf2-modulated genes in the human small airway epithelium is highly responsive to cigarette smoking. Mol Med 15: 203–219. 19593404 10.2119/molmed.2008.00130 PMC2707520

[47] Scholey JM, Anderson KV (2006) Intraflagellar transport and cilium-based signaling. Cell 125: 439–442. 16678091 10.1016/j.cell.2006.04.013

