# Supplementary material for: Intraflagellar Transport Gene Expression Associated with Short Cilia in Smoking and COPD
Source: PLoS One. 2014 Jan 20;9(1):e85453. doi: 10.1371/journal.pone.0085453 (PMC3896362; doi:10.1371/journal.pone.0085453)
Supplement: Figure S1 — Cilia length distributions obtained by measuring 100 cilia on 10 cells vs 500 cilia on 50 cells for each individual. (PDF) [file pone.0085453.s001.pdf]

Figure S1

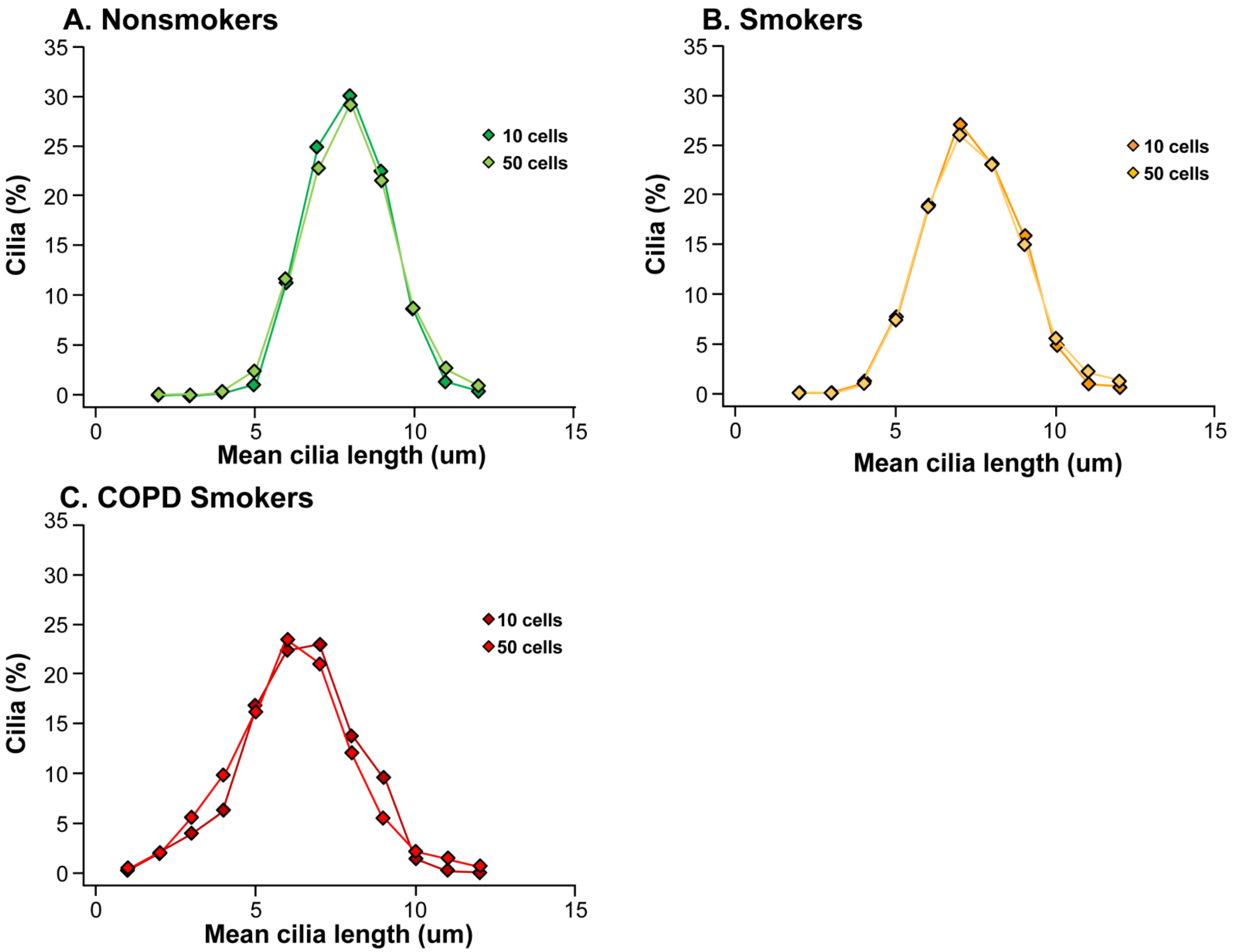

Supplemental Figure 1. Cilia length distributions obtained by measuring 100 cilia on 10 cells *vs* 500 cilia on 50 cells for each individual. The ordinate represents the % of individual cilia with a given length. The abscissa represents cilia length in µm. For each phenotype, a random subset of n=5 individuals were assessed. **A.** Cilia length distribution in nonsmokers. The dark green line represents the distribution obtained by measuring 100 cilia (10 cells) per individual. The light green line represents the distribution obtained by measuring 500 cilia (50 cells) per individual. **B.** Cilia length distribution in healthy smokers. The dark orange line represents the distribution obtained by measuring 100 cilia (10 cells) per individual. The light orange line represents the distribution obtained by measuring 500 cilia (50 cells) per individual. **C.** Cilia length distribution in COPD smokers. The dark red line represents the distribution obtained by measuring 100 cilia (10 cells) per individual. The light red line represents the distribution obtained by measuring 500 cilia (50 cells) per individual.
