# Supplementary material for: Intraflagellar Transport Gene Expression Associated with Short Cilia in Smoking and COPD
Source: PLoS One. 2014 Jan 20;9(1):e85453. doi: 10.1371/journal.pone.0085453 (PMC3896362; doi:10.1371/journal.pone.0085453)
Supplement: Figure S2 — Example of determination of mean cilia length in one individual. (PDF) [file pone.0085453.s002.pdf]

Figure S2

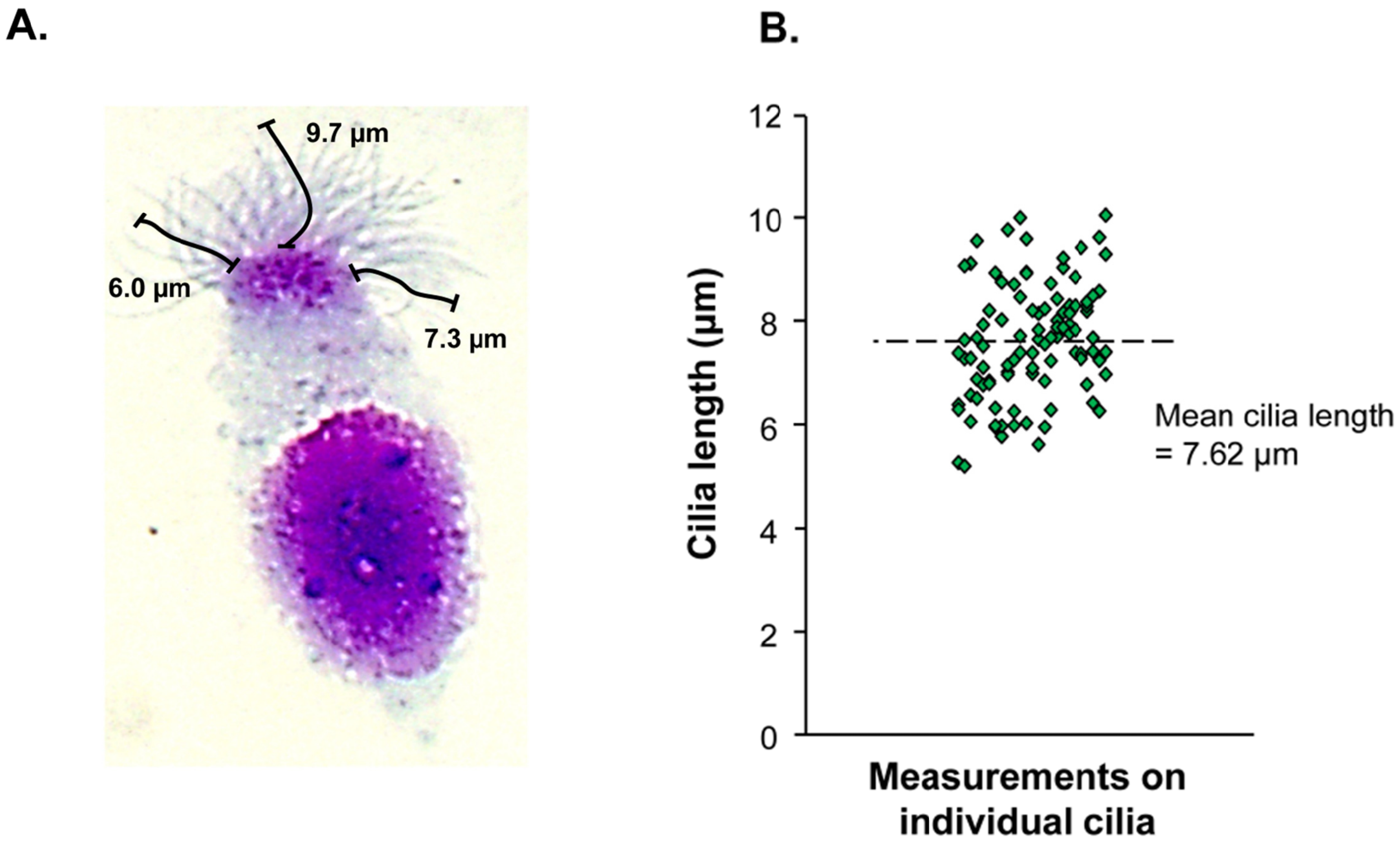

Supplemental Figure 2. Example of determination of mean cilia length in one individual. **A.** One ciliated cell is shown. Three examples of the length measurement of a single cilium are shown by black lines. For each individual, 10 cilia were measured on each of 10 ciliated cells. **B.** Distribution of cilia length for this individual. The ordinate shows cilia length. Each diamond represents the length of one cilium. The dashed line represents the mean cilia length for this individual.
