# Supplementary material for: Intraflagellar Transport Gene Expression Associated with Short Cilia in Smoking and COPD
Source: PLoS One. 2014 Jan 20;9(1):e85453. doi: 10.1371/journal.pone.0085453 (PMC3896362; doi:10.1371/journal.pone.0085453)
Supplement: Figure S3 — Variability of cilia length in the large (LAE) and small airway epithelium (SAE) of nonsmokers, healthy smokers and COPD smokers. (PDF) [file pone.0085453.s003.pdf]

Figure S3

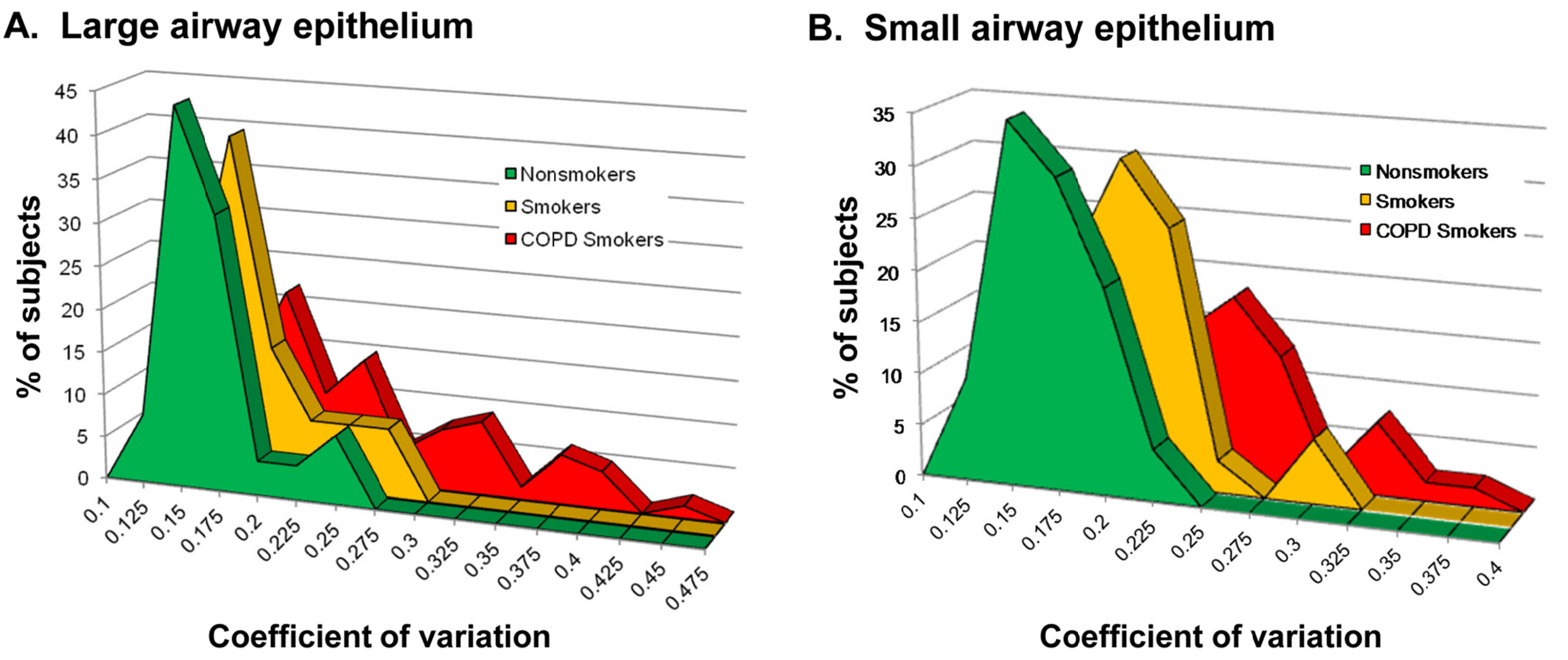

Supplemental Figure 3. Variability of cilia length in the large (LAE) and small airway epithelium (SAE) of nonsmokers, healthy smokers and COPD smokers. The distribution of the coefficient of variation in each phenotype is shown. The abscissa displays the coefficient of variation. The percent of subjects with that coefficient of variation is represented on the ordinate. Healthy nonsmokers are represented in green, healthy smokers in yellow, and smokers with COPD in red. There is a wider distribution of the coefficient of variation in healthy smokers compared to healthy nonsmokers, and an even wider distribution in smokers with COPD ($p<0.05$). **A.** Distribution of the coefficient of variation by phenotype in the large airway epithelium in healthy nonsmokers (n=25), healthy smokers (n=25), and smokers with COPD (n=70). **B.** Distribution of the coefficient of variation by phenotype in the small airway epithelium in healthy nonsmokers (n=20), healthy smokers (n=32), and smokers with COPD (n=56).
