# Supplementary material for: Intraflagellar Transport Gene Expression Associated with Short Cilia in Smoking and COPD
Source: PLoS One. 2014 Jan 20;9(1):e85453. doi: 10.1371/journal.pone.0085453 (PMC3896362; doi:10.1371/journal.pone.0085453)
Supplement: Table S1 — Inter-slide vs Inter-individual Variability in Cilia Length. (PDF) [file pone.0085453.s004.pdf]

Table S1. Inter-slide *vs* Inter-individual Variability in Cilia Length

Phenotype	Mean coefficient of variation per subject (inter-slide variability, %)[2]	Coefficient of variation across phenotype (inter-individual variability, %)[3]
Nonsmokers	5.8	9.7
Smokers	6.4	11.4
COPD Smokers	4.5	12.8

[1] Coefficient of variation (CoV) of mean cilia length was assessed per subject using LAE samples for a subset of nonsmokers (n=5), healthy smokers (n=5) and COPD smokers (n=5) and compared to the coefficient of variation across subjects of a given phenotype.

[2] CoV per subject was calculated by assessing cilia length on 5 slides prepared from each subject and calculating the CoV of mean cilia length for that individual. The mean CoV for the phenotype represents the average CoV value for n=5 subjects/phenotype assessed in this way.

[3] CoV across phenotype was calculated as the CoV of mean cilia length among all subjects for a given phenotype.
