# Supplementary material for: Intraflagellar Transport Gene Expression Associated with Short Cilia in Smoking and COPD
Source: PLoS One. 2014 Jan 20;9(1):e85453. doi: 10.1371/journal.pone.0085453 (PMC3896362; doi:10.1371/journal.pone.0085453)
Supplement: Table S2 — Expression of Intraflagellar Transport Genes in the Large and Small Airway Epithelium. (PDF) [file pone.0085453.s005.pdf]

Table S2. Expression of Intraflagellar Transport Genes in the Large and Small Airway Epithelium[1]

					Large airway epithelium[2]				Small airway epithelium[3]			
IFT component[4]	Probe set ID	General protein name	Gene symbol	Gene name	P call % nonsmoker[5]	P call % smoker	Fold-change[6]	p value[7]	P call % non-smoker	P call % smoker	Fold-change	p value
Anterograde IFT motor	**228680_at**	**Heterotrimeric kinesin-2**	**KIF3A**	**kinesin family member 3A**	**100**	**100**	**-1.25**	**1.7x10^{-2}**	**100**	**100**	**-1.12**	**2.4x10^{-2}**
	213623_at	Heterotrimeric kinesin-2	KIF3A	kinesin family member 3A	100	100	-1.27	7.6x10^{-2}	100	100	-1.08	3.5x10^{-1}
	225205_at	Heterotrimeric kinesin-2	KIF3B	kinesin family member 3B	100	100	-1.03	7.4x10^{-1}	100	100	-1.03	6.7x10^{-1}
	203943_at	Heterotrimeric kinesin-2	KIF3B	kinesin family member 3B	100	100	-1.01	9.5x10^{-1}	100	100	1.02	7.6x10^{-1}
	203333_at	Heterotrimeric kinesin-2	KIFAP3	kinesin-associated protein 3	100	100	-1.21	1.0x10^{-1}	100	100	1.09	2.2x10^{-1}
	222144_at	Homodimeric kinesin-2	KIF17	kinesin family member 17	24	13	-1.09	8.3x10^{-1}	34	33	-1.15	3.3x10^{-2}
Retrograde IFT motor	**1561939_at**	**Heavy chain**	**DYNC2H1**	**dynein, cytoplasmic 2, heavy chain 1**	**100**	**100**	**-1.81**	**9.3x10^{-3}**	**0**	**14**	**1.02**	**9.3x10^{-1}**
	**219469_at**	**Heavy chain**	**DYNC2H1**	**dynein, cytoplasmic 2, heavy chain 1**	**100**	**100**	**-1.24**	**1.0x10^{-1}**	**100**	**100**	**-1.15**	**3.6x10^{-2}**
	**224715_at**	**Intermediate chain**	**WDR34**	**WD repeat domain 34**	**100**	**100**	**-1.10**	**6.5x10^{-1}**	**100**	**100**	**-1.11**	**1.7x10^{-2}**
	1565149_at	Heavy chain	DYNC2H1	dynein, cytoplasmic 2, heavy chain 1	100	100	1.08	6.0x10^{-1}	100	100	-1.03	7.1x10^{-1}
	241757_x_at	Light intermediate chain	DYNC2LI1	dynein, cytoplasmic 2, light intermediate chain 1	100	100	-1.01	9.9x10^{-1}	100	100	-1.09	3.3x10^{-1}
	203762_s_at	Light intermediate chain	DYNC2LI1	dynein, cytoplasmic 2, light intermediate chain 1	100	100	-1.02	9.4x10^{-1}	100	100	1.02	7.6x10^{-1}
	203763_at	Light intermediate chain	DYNC2LI1	dynein, cytoplasmic 2, light intermediate chain 1	100	100	-1.11	3.7x10^{-1}	100	100	-1.08	3.2x10^{-1}
	1554324_s_at	Light intermediate chain	DYNC2LI1	dynein, cytoplasmic 2, light intermediate chain 1	86	77	1.00	9.9x10^{-1}	100	100	1.03	7.6x10^{-1}
	200703_at	Light chain	DYNLL1	dynein, light chain, LC8-type 1	100	100	-1.13	1.0x10^{-1}	100	100	1.04	3.8x10^{-1}
IFT complex A	**226195_at**	**IFT43**	**IFT43**	**intraflagellar transport 43 homolog (Chlamydomonas)**	**100**	**100**	**-1.29**	**4.0x10^{-2}**	**100**	**100**	**-1.18**	**1.1x10^{-2}**
	**226196_s_at**	**IFT43**	**IFT43**	**intraflagellar transport 43 homolog (Chlamydomonas)**	**100**	**100**	**-1.38**	**4.0x10^{-2}**	**100**	**100**	**-1.13**	**6.8x10^{-2}**
	**226889_at**	**IFT121, IFT122B**	**WDR35**	**WD repeat domain 35**	**100**	**100**	**-1.21**	**7.7x10^{-2}**	**100**	**100**	**-1.15**	**1.5x10^{-2}**
	**220917_s_at**	**IFT144**	**WDR19**	**WD repeat domain 19**	**100**	**100**	**-1.19**	**4.7x10^{-2}**	**100**	**100**	**-1.16**	**3.6x10^{-4}**
	**232163_at**	**IFT144**	**WDR19**	**WD repeat domain 19**	**100**	**100**	**-1.00**	**9.9x10^{-1}**	**100**	**100**	**-1.19**	**8.3x10^{-3}**

Table S2. Expression of Intraflagellar Transport Genes in the Large and Small Airway Epithelium[1] (cont. page 2)

					Large airway epithelium[2]				Small airway epithelium[3]			
IFT component[4]	Probe set ID	General protein name	Gene symbol	Gene name	P call % nonsmoker[5]	P call % smoker	Fold-change[6]	p value[7]	P call % non-smoker	P call % smoker	Fold-change	p value
	1568699_at	IFT43	IFT43	intraflagellar transport 43 homolog (Chlamydomonas)	95	90	-1.09	4.8x10^{-1}	100	97	-1.08	2.7x10^{-1}
	226890_at	IFT121, IFT122B	WDR35	WD repeat domain 35	100	100	-1.25	1.3x10^{-1}	100	100	1.08	2.5x10^{-1}
	220744_s_at	IFT122, IFT122A	IFT122	intraflagellar transport 122 homolog (Chlamydomonas)	100	100	-1.01	9.4x10^{-1}	100	100	-1.04	4.9x10^{-1}
	1563794_s_at	IFT122, IFT122A	IFT122	intraflagellar transport 122 homolog (Chlamydomonas)	52	48	-1.01	9.9x10^{-1}	97	93	-1.13	2.7x10^{-1}
	216678_at	IFT122, IFT122A	IFT122	intraflagellar transport 122 homolog (Chlamydomonas)	76	68	-1.09	6.9x10^{-1}	100	99	1.06	3.9x10^{-1}
	220064_at	IFT139	TTC21B	tetratricopeptide repeat domain 21B	100	100	-1.21	2.1x10^{-1}	100	100	-1.17	7.3x10^{-2}
	244585_at	IFT140	IFT140	intraflagellar transport 140 homolog (Chlamydomonas)	0	0	1.41	3.4x10^{-1}	0	0	1.44	6.9x10^{-2}
	204792_s_at	IFT140	IFT140	intraflagellar transport 140 homolog (Chlamydomonas)	100	100	1.04	8.6x10^{-1}	100	100	1.00	9.9x10^{-1}
	232844_at	IFT140	IFT140	intraflagellar transport 140 homolog (Chlamydomonas)	24	55	1.04	8.3x10^{-1}	100	100	-1.11	8.6x10^{-2}
	1555314_at	IFT144	WDR19	WD repeat domain 19	24	23	-1.34	4.6x10^{-1}	72	81	1.02	8.9x10^{-1}
IFT complex B	**215691_x_at**	**IFT25**	**HSPB11**	**heat shock protein family B (small), member 11**	**100**	**100**	**-1.04**	**7.4x10^{-1}**	**100**	**100**	**1.11**	**1.1x10^{-2}**
	**238494_at**	**IFT54**	**TRAF3IP1**	**TNF receptor-associated factor 3 interacting protein 1**	**100**	**100**	**-1.27**	**1.0x10^{-2}**	**100**	**100**	**-1.19**	**2.2x10^{-3}**
	**222520_s_at**	**IFT57**	**IFT57**	**intraflagellar transport 57 homolog (Chlamydomonas)**	**100**	**100**	**-1.43**	**9.3x10^{-3}**	**100**	**100**	**-1.20**	**4.5x10^{-3}**
	**1564231_at**	**IFT80**	**IFT80**	**intraflagellar transport 80 homolog (Chlamydomonas)**	**95**	**97**	**1.34**	**1.7x10^{-2}**	**100**	**100**	**-1.02**	**7.9x10^{-1}**
	**226324_s_at**	**IFT172**	**IFT172**	**intraflagellar transport 172 homolog (Chlamydomonas)**	**100**	**100**	**-1.23**	**2.3x10^{-2}**	**100**	**100**	**-1.18**	**3.6x10^{-4}**
	210312_s_at	IFT20	IFT20	intraflagellar transport 20 homolog (Chlamydomonas)	100	100	-1.20	9.0x10^{-2}	100	100	1.03	6.2x10^{-1}
	222742_s_at	IFT22	RABL5	RAB, member RAS oncogene family-like 5	100	100	-1.14	1.4x10^{-1}	100	100	1.02	7.6x10^{-1}
	218785_s_at	IFT22	RABL5	RAB, member RAS oncogene family-like 5	100	97	-1.17	1.3x10^{-1}	100	100	1.04	6.0x10^{-1}
	203960_s_at	IFT25	HSPB11	heat shock protein family B (small), member 11	100	100	-1.02	9.4x10^{-1}	100	100	1.10	1.4x10^{-1}
	214163_at	IFT25	HSPB11	heat shock protein family B (small), member 11	100	100	-1.01	9.7x10^{-1}	100	100	1.11	6.9x10^{-2}
	205037_at	IFT27	IFT27	intraflagellar transport 27 homolog (Chlamydomonas)	100	100	-1.20	1.4x10^{-1}	100	100	1.02	7.6x10^{-1}
	243812_at	IFT27	IFT27	intraflagellar transport 27 homolog (Chlamydomonas)	0	0	1.04	9.4x10^{-1}	0	4	1.11	4.9x10^{-1}
	213784_at	IFT27	IFT27	intraflagellar transport 27 homolog (Chlamydomonas)	100	100	-1.05	7.5x10^{-1}	100	100	-1.07	3.6x10^{-1}
	218483_s_at	IFT46	IFT46	intraflagellar transport 46 homolog	100	100	-1.03	8.3x10^{-1}	100	100	-1.02	7.6x10^{-1}

Table S2. Expression of Intraflagellar Transport Genes in the Large and Small Airway Epithelium[1] (cont. page 3)

					Large airway epithelium[2]				Small airway epithelium[3]			
IFT component[4]	Probe set ID	General protein name	Gene symbol	Gene name	P call % nonsmoker[5]	P call % smoker	Fold-change[6]	p value[7]	P call % non-smoker	P call % smoker	Fold-change	p value
				(Chlamydomonas)								
	218709_s_at	IFT52	IFT52	intraflagellar transport 52 homolog (Chlamydomonas)	100	100	-1.14	1.7x10^{-1}	100	100	-1.01	9.0x10^{-1}
	233532_x_at	IFT52	IFT52	intraflagellar transport 52 homolog (Chlamydomonas)	100	87	-1.14	3.4x10^{-1}	100	100	1.05	6.7x10^{-1}
	214458_at	IFT54	TRAF3IP1	TNF receptor-associated factor 3 interacting protein 1	90	71	-1.29	7.6x10^{-2}	100	94	-1.09	3.4x10^{-1}
	222519_s_at	IFT57	IFT57	intraflagellar transport 57 homolog (Chlamydomonas)	100	100	-1.42	5.2x10^{-2}	100	100	1.12	3.3x10^{-1}
	218100_s_at	IFT57	IFT57	intraflagellar transport 57 homolog (Chlamydomonas)	100	100	-1.13	3.0x10^{-1}	100	100	-1.03	6.0x10^{-1}
	213679_at	IFT70	TTC30A	tetratricopeptide repeat domain 30A	100	100	-1.18	3.0x10^{-1}	100	100	-1.03	7.6x10^{-1}
	1554588_a_at	IFT70	TTC30B	tetratricopeptide repeat domain 30B	100	100	-1.17	2.4x10^{-1}	100	100	1.02	7.9x10^{-1}
	243413_at	IFT70	TTC30B	tetratricopeptide repeat domain 30B	100	100	-1.12	3.4x10^{-1}	100	100	-1.02	7.9x10^{-1}
	61732_r_at	IFT74, IFT72	IFT74	intraflagellar transport 74 homolog (Chlamydomonas)	100	100	-1.14	3.3x10^{-1}	100	100	1.02	7.6x10^{-1}
	219174_at	IFT74, IFT72	IFT74	intraflagellar transport 74 homolog (Chlamydomonas)	100	100	-1.07	7.4x10^{-1}	100	100	1.03	7.5x10^{-1}
	226098_at	IFT80	IFT80	intraflagellar transport 80 homolog (Chlamydomonas)	100	100	-1.05	7.4x10^{-1}	100	100	1.07	8.1x10^{-2}
	1558956_s_at	IFT80	IFT80	intraflagellar transport 80 homolog (Chlamydomonas)	100	100	-1.00	9.9x10^{-1}	100	100	-1.01	8.4x10^{-1}
	240251_at	IFT80	IFT80	intraflagellar transport 80 homolog (Chlamydomonas)	19	23	1.62	1.2x10^{-1}	17	21	-1.22	3.8x10^{-1}
	219372_at	IFT81	IFT81	intraflagellar transport 81 homolog (Chlamydomonas)	100	100	-1.15	3.0x10^{-1}	100	100	-1.02	7.6x10^{-1}
	223736_at	IFT81	IFT81	intraflagellar transport 81 homolog (Chlamydomonas)	0	0	-1.18	7.4x10^{-1}	0	0	1.32	3.0x10^{-1}
	204703_at	IFT88	IFT88	intraflagellar transport 88 homolog (Chlamydomonas)	100	100	-1.16	9.7x10^{-2}	100	100	-1.06	1.5x10^{-1}
IFT complex A accessory	221964_at	IFT complex A accessory	TULP3	tubby like protein 3	95	87	-1.13	4.6x10^{-1}	100	100	-1.11	1.1x10^{-1}
	205854_at	IFT complex A accessory	TULP3	tubby like protein 3	100	100	-1.08	3.8x10^{-1}	100	100	-1.08	1.2x10^{-1}
IFT complex B accessory	**204576_s_at**	**IFT complex B accessory**	**CLUAP1**	**clusterin associated protein 1**	**100**	**100**	**-1.36**	**1.1x10^{-2}**	**100**	**100**	**-1.09**	**2.9x10^{-1}**
	**204577_s_at**	**IFT complex B accessory**	**CLUAP1**	**clusterin associated protein 1**	**100**	**100**	**-1.29**	**4.0x10^{-2}**	**100**	**100**	**-1.14**	**1.3x10^{-2}**
	**233999_s_at**	**IFT complex B accessory**	**TTC26**	**tetratricopeptide repeat domain 26**	**100**	**100**	**-1.40**	**5.2x10^{-2}**	**100**	**100**	**1.27**	**1.3x10^{-2}**
	**219758_at**	**IFT complex B accessory**	**TTC26**	**tetratricopeptide repeat domain 26**	**100**	**100**	**-1.22**	**1.4x10^{-1}**	**100**	**100**	**1.17**	**9.0x10^{-3}**
	1554672_at	IFT complex B accessory	TTC26	tetratricopeptide repeat domain 26	67	71	-1.06	8.3x10^{-1}	93	94	1.17	2.5x10^{-1}

Table S2. Expression of Intraflagellar Transport Genes in the Large and Small Airway Epithelium[1] (cont. page 4)

					Large airway epithelium[2]				Small airway epithelium[3]			
IFT component[4]	Probe set ID	General protein name	Gene symbol	Gene name	P call % nonsmoker[5]	P call % smoker	Fold-change[6]	p value[7]	P call % non-smoker	P call % smoker	Fold-change	p value
	235949_at	IFT complex B accessory	TTC26	tetratricopeptide repeat domain 26	100	100	-1.32	$5.2x10^{-2}$	100	100	-1.03	$7.6x10^{-1}$
BBSome	**230697_at**	**BBS5**	**BBS5**	**Bardet-Biedl syndrome 5**	**100**	**90**	**-1.34**	**$1.7x10^{-2}$**	**100**	**100**	**1.10**	**$2.5x10^{-1}$**
	**209958_s_at**	**BBS9**	**BBS9**	**Bardet-Biedl syndrome 9**	**100**	**94**	**-1.30**	**$4.7x10^{-2}$**	**100**	**96**	**-1.07**	**$2.9x10^{-1}$**
	**213220_at**	**BBIP10**	**BBIP1**	**BBSome interacting protein 1**	**100**	**100**	**-1.19**	**$2.0x10^{-1}$**	**100**	**100**	**1.19**	**$2.6x10^{-5}$**
	222643_s_at	BBS1	BBS1	Bardet-Biedl syndrome 1	90	65	-1.35	$7.6x10^{-2}$	21	56	1.29	$1.3x10^{-1}$
	218471_s_at	BBS1	BBS1	Bardet-Biedl syndrome 1	100	100	-1.08	$5.1x10^{-1}$	100	100	1.01	$8.9x10^{-1}$
	229142_s_at	BBS1	BBS1	Bardet-Biedl syndrome 1	0	6	-1.11	$7.4x10^{-1}$	0	0	1.08	$7.6x10^{-1}$
	223227_at	BBS2	BBS2	Bardet-Biedl syndrome 2	100	100	-1.09	$2.9x10^{-1}$	100	100	-1.06	$2.7x10^{-1}$
	212745_s_at	BBS4	BBS4	Bardet-Biedl syndrome 4	100	100	-1.19	$1.3x10^{-1}$	100	100	1.07	$2.7x10^{-1}$
	212744_at	BBS4	BBS4	Bardet-Biedl syndrome 4	100	100	-1.08	$4.8x10^{-1}$	100	100	1.05	$3.2x10^{-1}$
	219688_at	BBS7	BBS7	Bardet-Biedl syndrome 7	95	84	1.02	$9.5x10^{-1}$	100	100	1.11	$8.3x10^{-2}$
	235007_at	BBS7	BBS7	Bardet-Biedl syndrome 7	100	100	-1.12	$3.4x10^{-1}$	100	100	-1.06	$3.8x10^{-1}$
	226120_at	BBS8	TTC8	tetratricopeptide repeat domain 8	100	100	-1.06	$6.1x10^{-1}$	100	100	1.02	$7.6x10^{-1}$
	37549_g_at	BBS9	BBS9	Bardet-Biedl syndrome 9	100	100	-1.10	$4.8x10^{-1}$	100	100	-1.03	$7.6x10^{-1}$
	37547_at	BBS9	BBS9	Bardet-Biedl syndrome 9	100	100	-1.07	$7.4x10^{-1}$	100	100	1.01	$8.9x10^{-1}$
	216239_at	BBS9	BBS9	Bardet-Biedl syndrome 9	48	29	1.19	$7.5x10^{-1}$	48	36	-1.05	$8.4x10^{-1}$
	1555555_at	BBS9	BBS9	Bardet-Biedl syndrome 9	29	32	1.05	$9.4x10^{-1}$	69	64	-1.07	$6.7x10^{-1}$
	228993_s_at	BBIP10	BBIP1	BBSome interacting protein 1	100	100	1.06	$7.4x10^{-1}$	100	100	1.07	$1.4x10^{-1}$
	213224_s_at	BBIP10	BBIP1	BBSome interacting protein 1	100	100	-1.21	$9.0x10^{-2}$	100	100	1.00	$9.3x10^{-1}$
	232885_at	BBIP10	BBIP1	BBSome interacting protein 1	95	100	1.24	$2.3x10^{-1}$	100	100	-1.03	$7.6x10^{-1}$
	232910_at	BBIP10	BBIP1	BBSome interacting protein 1	10	23	1.07	$8.3x10^{-1}$	34	31	1.09	$2.7x10^{-1}$

[1] Data obtained using Affymetrix U133 Plus 2.0 microarray chips. Significant genes in either LAE or SAE are shown in **bold** font.
[2] 3rd-4th order airway epithelium obtained by bronchoscopic brushing.
[3] 10th-12th order airway epithelium obtained by bronchoscopic brushing.
[4] IFT components and genes adapted from Ishikawa et al, Nat Rev Mol Cell Biol, 2011;12:222-234.
[5] P call % represents the % of samples for which the Affymetrix detection call was Present. Probe set IDs with a P call of ≥ 20% were considered to be expressed.
[6] Fold-change represents the ratio of mean expression in healthy smokers to mean expression in healthy nonsmokers, with positive values representing genes up-regulated in smokers and negative values representing genes down-regulated in smokers.
[7] p values are corrected for multiple testing using the Benjamini-Hochberg method. $p \leq 0.05$ was considered to be significant.
