# Supplementary material for: Intraflagellar Transport Gene Expression Associated with Short Cilia in Smoking and COPD
Source: PLoS One. 2014 Jan 20;9(1):e85453. doi: 10.1371/journal.pone.0085453 (PMC3896362; doi:10.1371/journal.pone.0085453)
Supplement: Table S3 — TaqMan Confirmation of Significantly Modified Intraflagellar Transport Genes. (PDF) [file pone.0085453.s006.pdf]

Table S3. TaqMan Confirmation of Significantly Modified Intraflagellar Transport Genes[1]

Gene symbol	Fold-change[2]	p value[3]
KIF3A	-1.63	$7.74x10^{-2}$
TRAF3IP1	-1.48	$2.72 x10^{-2}$
IFT57	-1.42	$3.57 x10^{-2}$
IFT172	-1.65	$3.42 x10^{-3}$
CLUAP1	-1.29	$1.59 x10^{-1}$
DYNC2H1	-1.63	$1.52 x10^{-2}$
IFT43	-1.38	$1.23 x10^{-2}$

[1] Data obtained via TaqMan RT-PCR on LAE samples from the LAE microarray set of subjects.

[2] Fold-change represents the ratio of mean expression in healthy smokers to mean expression in healthy nonsmokers, with positive values representing genes up-regulated in smokers and negative values representing genes down-regulated in smokers.

[3] p values were calculated using Student's t test.
