# Supplementary material for: Intraflagellar Transport Gene Expression Associated with Short Cilia in Smoking and COPD
Source: PLoS One. 2014 Jan 20;9(1):e85453. doi: 10.1371/journal.pone.0085453 (PMC3896362; doi:10.1371/journal.pone.0085453)
Supplement: Text S1 — Supplemental Methods. (PDF) [file pone.0085453.s007.pdf]

Supplemental Methods

Inclusion/Exclusion Criteria

Healthy nonsmokers

Inclusion criteria

- Capable of providing informed consent
- Men and women, age 18 or older
- Never-smokers by history, with current smoking status validated by the absence of nicotine metabolites in urine [1]
- Good overall health without history of chronic lung disease, including asthma, and without recurrent or recent (within 3 months) acute pulmonary disease
- Normal physical examination
- Normal routine laboratory evaluation, including general hematologic studies, general serologic/immunologic studies, general biochemical analyses, and urine analysis
- Negative HIV serology
- Normal chest X-ray (PA and lateral)
- Normal electrocardiogram
- Women - not pregnant
- No history of allergies to medications to be used in the bronchoscopy procedure
- Not taking any medications relevant to lung disease or having an effect on the airway epithelium
- Willingness to participate in the study

Exclusion criteria

- Unable to meet the inclusion criteria
- Pregnancy
- Current active infection or acute illness of any kind
- Current alcohol or drug abuse
- Evidence of malignancy within the past 5 years

Healthy smokers

Inclusion criteria

- Capable of providing informed consent
- Men and women, age 18 or older
- Current daily smokers with any number of pack-yr, validated by any of the following: urine nicotine >30 ng/ml or urine cotinine >50 ng/ml [1]
- Good overall health without history of chronic lung disease, including asthma, and without recurrent or recent (within 3 months) acute pulmonary disease
- Normal physical examination
- Normal routine laboratory evaluation, including general hematologic studies, general serologic/immunologic studies, general biochemical analyses, and urine analysis
- Negative HIV serology
- Normal chest X-ray (PA and lateral)

- Normal electrocardiogram
- Women - not pregnant
- No history of allergies to medications to be used in the bronchoscopy procedure
- Not taking any medications relevant to lung disease or having an effect on the airway epithelium
- Willingness to participate in the study

Exclusion criteria
- Unable to meet the inclusion criteria
- Pregnancy
- Current active infection or acute illness of any kind
- Current alcohol or drug abuse
- Evidence of malignancy within the past 5 years

Smokers with COPD

Inclusion criteria
- Capable of providing informed consent
- Men and women, age 18 or older
- Current daily smokers with any number of pack-yr, validated by any of the following: urine nicotine >30 ng/ml or urine cotinine >50 ng/ml [1]
- Meeting GOLD stages I-IV criteria for chronic obstructive lung disease (COPD) based on post-bronchodilator spirometry
- Taking any or no pulmonary-related medication, including beta-agonists, anticholinergics, or inhaled corticosteroids
- Normal routine laboratory evaluation, including general hematologic studies, general serologic/immunologic studies, general biochemical analyses, and urine analysis
- Women - not pregnant
- Negative HIV serology
- Normal electrocardiogram (sinus bradycardia, premature atrial contractions are permissible)
- All individuals have chest X-ray (PA and lateral)
- No history of allergies to medications to be used in the bronchoscopy procedure
- Willingness to participate in the study

Exclusion criteria
- Unable to meet the inclusion criteria
- Individuals in whom participation in the study would compromise the normal care and expected progression of their disease
- Current active infection or acute illness of any kind
- Current alcohol or drug abuse
- Evidence of malignancy within the past 5 years

Quantitative Analysis of Emphysema

A subset of subjects of all phenotypes had high resolution chest computed tomography (HRCT) scans acquired according to a standard research protocol as follows. Scans were acquired on a GE 64-row scanner in the supine position from lung apex to costophrenic angle using tube voltage 120 kV and tube current 130 mA with 0.625 mm collimation. Emphysema was quantified using EmphylxJ software (Vancouver, Canada) and was defined as the % of lung volume for which the attenuation value was ≤ -950 Hounsfield units.

Sample Collection and Preparation

Large airway epithelium (LAE) and small airway epithelium (SAE) were obtained by brushing the epithelium as previously described. Cells were detached from the brush by flicking into 5 mL of ice cold bronchial epithelial cell basal medium (BEBM, Lonza, Allendale, NJ). Cells were applied to microscope slides using centrifugal force (Cytospin 11, Shandon Instruments, Pittsburgh, PA), air-dried, fixed in 4% paraformaldehyde in PBS (Gibco, Grand Island, NY), and stained using Diff-Quik (Dade Behring, Deerfield, IL). RNA was processed for microarray analysis as previously described [2].

Validation of Method for Determining Mean Cilia Length

To assess whether 100 cilia were a representative sample for an individual, in a subset of subjects (n=5 nonsmokers, n=5 healthy smokers, n=5 COPD smokers), 500 cilia were measured (10 cilia on each of 50 cells). A mean cilia length for each individual was calculated using the 50 cell set (500 cilia), and this mean was then compared to the mean cilia length of the 10 cell set (100 cilia; Figure S1). These mean lengths were not significantly different (all $p>0.05$) and the distribution curves were visually similar, suggesting that evaluating 100 cilia adequately represented the population of cilia for an individual.

To assess whether one slide adequately represented the population of cilia for one

subject, in a subset of subjects (n=5 nonsmokers, n=5 healthy smokers, n=5 COPD smokers), 100 cilia (10 cilia on each of 10 cells) were measured on 5 slides per subject. The mean cilia length was calculated for each slide and coefficient of variation calculated for each subject, and this was compared to the coefficient of variation within each phenotype. For each phenotype, the mean coefficient of variation across 5 slides for one subject was less than the coefficient of variation across subjects of that phenotype (Table S1), suggesting that the variability in cilia length among slides from one individual was less significant than the variability among individuals.

RNA and Microarray Processing

Gene expression was evaluated using the HG-U133 Plus 2.0 array (Affymetrix, Santa Clara, CA), which includes probes for >47,000 transcripts genome-wide. Total RNA was extracted using a modified version of the TRIzol method (InVitrogen, Carlsbad, CA), in which RNA is purified directly from the aqueous phase (RNeasy MinElute RNA purification kit, Qiagen, Valencia, CA). RNA samples were stored in RNAsecure (Ambion, Austin, TX) at -80°C. RNA integrity was confirmed by running an aliquot of each RNA sample on an Agilent Bioanalyzer (Agilent Technologies, Palo Alto, CA) and a NanoDrop ND-1000 spectrophotometer (NanoDrop Technologies, Wilmington, DE) was used to determine the concentration. Double stranded cDNA was synthesized from 1-2 μg total RNA using the GeneChip One-Cycle cDNA Synthesis Kit, followed by cleanup with GeneChip Sample Cleanup Module, in vitro transcription (IVT) reaction with the GeneChip IVT Labeling Kit, and cleanup and quantification of the biotin-labeled cDNA yield by spectrophotometry. All kits were from Affymetrix (Santa Clara, CA). All HG-U133 Plus 2.0 microarrays were processed according to Affymetrix protocols, hardware and software, including being processed by the Affymetrix fluidics station 450 and hybridization oven 640 and scanned with an Affymetrix Gene Array

Scanner 3000 7G. Overall microarray quality was verified by the following criteria [3-6]: (1) 3'/5' ratio for GAPDH <3; and (2) scaling factor <10.0.

Microarray Data Analysis

Microarray data were processed using the MAS5 algorithm (Affymetrix Microarray Suite Version 5 software), which takes into account the perfect match and mismatch probes. MAS5-processed data were normalized using GeneSpring by setting measurements <0.01 to 0.01 and by normalizing per chip to the median expression value on the array and per gene to the median expression value for each gene across all arrays. Gene expression was evaluated for IFT-related genes. Genes with an Affymetrix P call in ≥20% of samples were considered to be expressed. Differences in expression were evaluated by Student's t test. A p value <0.05 using a Benjamini-Hochberg correction to limit the false positive rate was considered to represent a significant difference in expression between groups. The fold-change (FC) represented the ratio of mean expression in healthy smokers to mean expression in nonsmokers, with positive FC values representing increased expression and negative values decreased expression in smokers. Gene expression was assessed in the independent data sets from the LAE and SAE. To evaluate whether IFT gene expression was related to % ciliated cells in the airway epithelium, R^2 and p values were calculated for the correlation between the 8 genes found to be significant in both data sets and the % ciliated cells in the sample. In the LAE there were no significant correlations (all p>0.08). In the SAE, significant correlations were observed for all 8 genes (p<0.05); however, the observed R^2 values were small (all <0.26), indicating that the contribution of % ciliated cells to the observed expression was small. Finally, gene expression was evaluated using the criteria described above in the SAE of COPD smokers *vs* healthy smokers.

TaqMan Real-Time Reverse Transcriptase–PCR

TaqMan real-time reverse transcriptase (RT)-PCR was used to confirm the 8 gene expression differences between groups that were observed by microarray analysis, using available LAE samples from the cohort used in the microarray analysis. The n for each assay varied based on the number of samples available (nonsmokers, n=8-14 and healthy smokers, n=8-15). Premade TaqMan Gene Expression Assays were obtained from Applied Biosystems (Foster City, CA). All samples were assessed in duplicate at two different cDNA concentrations to ensure that amplification efficiency for the reference gene and gene of interest was the same. All PCR reactions were run in an Applied Biosystems Sequence Detection System 7500. Relative mRNA abundance was calculated using 18S ribosomal RNA as a reference gene.

Statistical Analysis

Subject demographics were assessed by chi-square for categorical variables and analysis of variance (ANOVA) for continuous variables. The difference in mean cilia length between phenotypes was assessed by ANOVA with *post hoc* pairwise testing where appropriate and p values <0.05 were taken to mean that the populations were significantly different. The coefficient of variation was calculated for each subject, and a Kruskal-Wallis one-way analysis of variance was used to determine if the distributions of the coefficients of variation for each phenotype were significantly different. Correlations between mean cilia length and demographic and lung function parameters as well as gene expression levels were done using ANOVA for categorical variables and linear regression for continuous variables as well as multivariate regression analysis (Stata 10.1, StataCorp, College Station, TX) and p<0.05 was considered to represent a statistically significant correlation.

To assess whether the decrease in cilia length in smokers was relatively uniform (all cilia shortened to the same extent) or more variable (some cilia remaining long while others are shortened), a coefficient of variation (CoV) in mean cilia length was calculated for each

individual, and the distribution of CoV values was assessed for each phenotype. The distributions of CoV values for each phenotype were significantly different ($p<0.004$ for LAE and SAE, Figure S3A,B). In the LAE, 16% of the healthy smokers and 40% of smokers with COPD had a CoV of mean cilia length exceeding the 95th percentile among nonsmokers. In the SAE, 41% of the healthy smokers and 57% of smokers with COPD had a CoV of mean cilia length exceeding the 95th percentile among nonsmokers.

Supplemental References

1. Moyer,TP, Charlson JR, Enger RJ, Dale LC, Ebbert JO, Schroeder DR, Hurt RD. Simultaneous analysis of nicotine, nicotine metabolites, and tobacco alkaloids in serum or urine by tandem mass spectrometry, with clinically relevant metabolic profiles. *Clin Chem* 2002; 48: 1460-1471

2. Tilley,AE, O'Connor TP, Hackett NR, Strulovici-Barel Y, Salit J, Amoroso N, Zhou XK, Raman T, Omberg L, Clark A, Mezey J, Crystal RG. Biologic phenotyping of the human small airway epithelial response to cigarette smoking. *PLoS One* 2011; 6: e22798

3. Jung,M, Ramankulov A, Roigas J, Johannsen M, Ringsdorf M, Kristiansen G, Jung K. In search of suitable reference genes for gene expression studies of human renal cell carcinoma by real-time PCR. *BMC Mol Biol* 2007; 8: 47-

4. Stan,AD, Ghose S, Gao XM, Roberts RC, Lewis-Amezcua K, Hatanpaa KJ, Tamminga CA. Human postmortem tissue: what quality markers matter? *Brain Res* 2006; 1123: 1-11

5. Thompson,KL, Pine PS, Rosenzweig BA, Turpaz Y, Retief J. Characterization of the effect of sample quality on high density oligonucleotide microarray data using progressively degraded rat liver RNA. *BMC Biotechnol* 2007; 7: 57

6. Tumor Analysis Best Practices Working Group. Expression profiling--best practices for data generation and interpretation in clinical trials. *Nat Rev Genet* 2004; 5: 229-237
